# Phenotyping senescent mesenchymal stromal cells using AI image translation

**DOI:** 10.1016/j.crbiot.2023.100120

**Published:** 2023-02-01

**Authors:** Leya Weber, Brandon S. Lee, Sara Imboden, Cho-Jui Hsieh, Neil Y.C. Lin

**Affiliations:** a Department of Mechanical and Aerospace Engineering, University of California, Los Angeles 90095, CA, United States; b Department of Bioengineering, University of California, Los Angeles 90095, CA, United States; c Department of Computer Science, University of California, Los Angeles 90095, CA, United States; d California NanoSystems Institute, University of California, Los Angeles 90095, CA, United States; e Jonsson Comprehensive Cancer Center, University of California, Los Angeles 90095, CA, United States; f Institute for Quantitative and Computational Biosciences, University of California, Los Angeles 90095, CA, United States; g Broad Stem Cell Center, University of California, Los Angeles 90095, CA, United States

**Keywords:** MSC phenotyping, Senescence, AI image translation, Cell manufacturing

## Abstract

Mesenchymal stromal cells (MSCs) offer promising potential in biomedical research, clinical therapeutics, and immunomodulatory therapies due to their ease of isolation and multipotent, immunoprivileged, and immunosuppersive properties. Extensive efforts have focused on optimizing the cell isolation and culture methods to generate scalable, therapeutically-relevant MSCs for clinical applications. However, MSC-based therapies are often hindered by cell heterogeneity and inconsistency of therapeutic function caused, in part, by MSC senescence. As such, noninvasive and molecular-based MSC characterizations play an essential role in assuring the consistency of MSC functions. Here, we demonstrated that AI image translation algorithms can effectively predict immunofluorescence images of MSC senescence markers from phase contrast images. We showed that the expression level of senescence markers including senescence-associated beta-galactosidase (SABG), p16, p21, and p38 are accurately predicted by deep-learning models for Doxorubicin-induced MSC senescence, irradiation-induced MSC senescence, and replicative MSC senescence. Our AI model distinguished the non-senescent and senescent MSC populations and simultaneously captured the cell-to-cell variability within a population. Our microscopy-based phenotyping platform can be integrated with cell culture routines making it an easily accessible tool for MSC engineering and manufacturing.

## 1. Introduction

Human mesenchymal stromal cells (MSCs) are multipotent stem cells capable of self-renewal and differentiation into various cell types including adipocytes, chondrocytes, and osteocytes(Oja et al., 2018; Entzeroth et al., 2009; Zhou et al., 2020; Turinetto et al., 2016; Uder et al., 2018; Bertolo et al., 2019; Yang et al., 2018; Patel et al., 2013). MSC functions, including proliferation, differentiation multipotency, paracrine effect, and immunomodulatory activities, make them a valuable therapeutic agent for treating immune system disorders (Newman et al., 2009; Liu et al., 2020; Zhou et al., 2020; Wang et al., 2016), bone and cartilage injury, as well as cardiovascular(Liu et al., 2020; Zhou et al., 2020) or neurological diseases and damages (Liu et al., 2020; Zhou et al., 2020; Azari et al., 2010). While recent lab-based studies and clinical trials have demonstrated promising MSC therapeutic applications, (Bertolo et al., 2019; Uder et al., 2018; Turinetto et al., 2016; Imboden et al., 2021; Zhou et al., 2020) practical translation remains challenging due to low reproducibility of trial outcomes(Marklein et al., 2019; Marklein et al., 2018; Zhou et al., 2020). Specifically, MSC functional heterogeneity currently presents a major technical challenge in developments towards MSC therapeutic applications, such as decreasing immunomodulatory capacity and differentiation potential(Marklein et al., 2018; Mortensen et al., 2022).

Furthermore, MSC functions usually decay quickly during *in vitro* expansion, which presents a critical manufacturing challenge to acquire enough cells for clinical administration (Zhou et al., 2020). Additionally, all primary MSCs can only undergo a limited number of divisions due to telomere shortening and eventually enter replicative senescence (Debacq-Chainiaux et al., 2016; Zhou et al., 2020; Oja et al., 2018; Turinetto et al., 2016; Bertolo et al., 2019; Zhou et al., 2020). Such an aging process is often accelerated *in vitro* because most culture environments and signaling factors are drastically different from that in native tissues, resulting in excessive intracellular stress and misregulated autophagy(Zhou et al., 2020; Rubin, 2002). In addition, constant exposure to endogenous or exogenous stress factors can cause damage to the cells and also induce subsequent irreversible cell cycle arrest(Turinetto et al., 2016; Kamal et al., 2020; Ko et al., 2012). This stress-induced aging, termed premature senescence(Debacq-Chainiaux et al., 2016), can be triggered by various stimuli including reactive oxygen species(Kamal et al., 2020; Zhou et al., 2020; Ko et al., 2012), mechanical stress(Zhou et al., 2020), hypoxia(Zhou et al., 2020), chemotherapeutic(Kamal et al., 2020) or ionizing radiation(Zhou et al., 2020; Kamal et al., 2020; Zhou et al., 2020; Ko et al., 2012). *In vitro* MSC senescence has been shown to be largely responsible for compromised therapeutic functions(Bertolo et al., 2019; Turinetto et al., 2016). Developing strategies to combat *in vitro* senescence and ensure effective cell therapy outcomes has been difficult to accomplish(Turinetto et al., 2016; Phinney, 2012) in which major challenges are associated with the inability to comprehensively assess properties of live MSCs since current characterization assessments are either relatively non-specific, time-consuming, or invasive(Bertolo et al., 2019; Dwarshuis et al., 2017; Rivière and Roy, 2017).

Despite these challenges, pioneering works have developed various methods to assess MSC senescence. Currently, standard senescence evaluation involves staining for senescence markers such as senescence-associated beta-galactosidase (SABG)(Roger et al., 2021; Bertolo et al., 2019), oxidative stress markers (Vono et al., 2018), and DNA damage markers(Bertolo et al., 2019) to subsequently perform fluorescence-based characterizations (e.g., microscopy or cytometry(Alessio et al., 2015; Bellotti et al., 2016; O’Hagan-Wong et al., 2016)). While this approach provides quantitative molecule-based assessments and resolves intra-population cell heterogeneity, it usually relies on immunostaining. This process thus requires cell fixation, which invasively perturbs cells and can be time consuming(Bertolo et al., 2019; Christiansen et al., 2018). Moreover, the evolution of marker expression cannot be tracked over time for the same sample due to the fixation requirement. Recently, a few noninvasive phenotypic senescence markers have been introduced(Bertolo et al., 2019; Lin et al., 2019; Oja et al., 2018). Specifically, studies have shown that MSC size and shape are related to telomere length and immunosuppressive capacity(Marklein et al., 2019), suggesting that MSC morphology can be used as a senescence marker surrogate(Marklein et al., 2019). Futhermore, flow cytometry experiments have similarly shown that autofluorescence can be utilized as an *in vitro* marker (Bertolo et al., 2019). Despite such significant progress, the establishment of a method that can non-invasively report expression of molecule-based markers (e.g., SABG and DNA damage markers) (Simmons and Torok-Storb, 1991; Ode et al., 2011; Lo Surdo and Bauer, 2012) in live MSCs remains elusive.

AI image translation is a powerful tool for analyzing and enhancing microscopy data (Christiansen et al., 2018; Ounkomol et al., 2018; Jin et al., 2017; Wang et al., 2019; Weigert et al., 2018; Goldschmidt et al., 1996; Bermudez et al., 2022; Imboden et al., 2021; Kim et al., 2019; El Achi and Khoury, 2020; Raila et al., 2022). Visual features that are difficult to detect using traditional analyses can be uncovered by deep learning(Marklein et al., 2019; Hennig et al., 2017). For example, features beyond the diffraction limit can be extracted from conventional fluorescent images using an AI model with super-resolution microscopy data(Imboden et al., 2021). Image translation algorithms have also been used to perform *in silico* histological staining and organelle labeling(Christiansen et al., 2018). In cell research, image translation models have been shown to correctly predict the expression level of essential surface markers(Hennig et al., 2017; Christiansen et al., 2018; Rivenson et al., 2019). Here, we demonstrated that such an AI image labeling technique can be employed to non-invasively and scalably label extra-cellular marker expression of live senescent MSCs in real-time. We trained our deep learning neural networks using paired phase contrast and immunofluorescent images. We showed that a fully trained AI model can accurately predict the expression level of common senescence markers, including SABG, p16, p21, and p38 in both stress-induced and replication-induced senescent cells.

## 2. Materials and methods

### Cell culture.

Immortalized human adipose-derived MSCs (ATCC, SCRC-4000) and human bone marrow-derived MSCs (Lonza, PT-2501) were cultured according to previous published work in high glucose Dulbecco’s Modified Eagle Medium (Gibco, 4.5 g/L glucose, 500 mL) supplemented with 10% fetal bovine serum (Gibco) and 1% Penicillin-Streptomycin (Gibco) (ref) or in StemFit for MSC (Ajinomoto) and 1% Penicillin-Streptomycin (Gibco). After thawing, MSCs were seeded into tissue culture flasks at a density of 5k cells/cm^2^. MSC culture media was replaced every 48 hours. At 80% confluency subculture was performed, in which cells were washed with 1X PBS−/− (without calcium or magnesium) twice, following incubation with 0.5% Trypsin-EDTA at 37°C leading to cell detachment. After adding high glucose DMEM cell solution was transferred to a fresh tube and centrifuged at 300g for 3 minutes. Cells were resuspended in warmed culture media and reseeded at a density of 5k cells/cm^2^. For all experiments, cells were seeded into 4-chamber microscope slides (Ibidi, 80446) at a seeding density of 5k cells/cm^2^.

### Senescence marker immunostaining.

All antibodies were purchased from Cell Signaling Technology (p16Ink4a, p21 Waf1/Cip1, p38 MAPK antibody, CD44). Fixation and immunostaining of samples was performed as previously described (Imboden et al., 2021). Briefly, samples were washed with 1X PBS+/+ (with calcium and magnesium) twice followed by fixing with 4% PFA (ThermoFisher Scientific, 28908). After 5 min of incubation, the samples were washed again with 1X PBS+/+ twice. For staining, a blocking buffer solution prepared of 2% donkey serum (Sigma-Aldrich, D9663-10ML) and 0.5% Triton X-100 (Sigma-Aldrich, T8787-50ML) was added to each sample and incubated for 30 min. After the second washing step with 1X PBS+/+ staining solution of primary antibodies was added and incubated overnight at 4°C. Samples are washed again with 1X PBS+/+ followed by incubation of 30 minutes with second staining solution (secondary antibodies + NucBlue). Lastly, samples were washed twice with 1X PBS+/+ and additionally, 0.1% Tween (Sigma-Aldrich, P9416-50ML) was added for storage. The SA-*β*-gal staining (CellEvent, Invitrogen, C10850) was conducted using the vendor’s protocol. In summary, cells were washed twice with 1X PBS+/+ , followed by fixing with 4% PFA for 30 min at room temperature. After washing the cells twice with 1X PBS+/+ , the sample was incubated with the SA-*β*-gal working solution for 2 h at room temperature.

### Imaging and image analysis.

All stained samples were imaged with an inverted fluorescent microscope (Etaluma LS720, Lumaview 720/600-series software) using a 20× objective (Olympus, LCACHN 20 XIPC). For each imaging channel (i.e., phase-contrast, 405 nm, 488 nm, and 597 nm), approximately 600 images were acquired with a field of view 0.38 mm × 0.38 mm. To perform the single-cell measurement, polygon selection tool in Fiji ImageJ was used to outline the single cells of immunofluorescence and the AI-predicted images. The cells were outlined manually to ensure the accuracy of outlining. For each tested condition, we analyzed 30 cells. Overall pixel-intensity is then used to analyze and evaluate prediction accuracy of the AI model.

### Doxorubicin and irradiation treatments.

To conduct the Doxorubicin treatment, ad-MSCs at a confluency ~ 80% were incubated with 0.5 *μ*M Doxorubicin for 48 hours at 37°C, 5% CO_2_. The cell media were then discarded and the samples were incubated in fresh culture media for another 24 hours for recovery. To perform the irradiation treatment, ad-MSCs (~ 80% confluency) were irradiated with X-ray (Rad-Source, RS2000) with a dose rate of 8.5 Gy/min and a dose concentration of 200 Gy, followed by replacing the supernatant with fresh cell medium. After treatment, the samples were incubated for 8 days where the culture medium was replaced every other day.

### Deep learning model development and AI training.

**Fig. 1.**
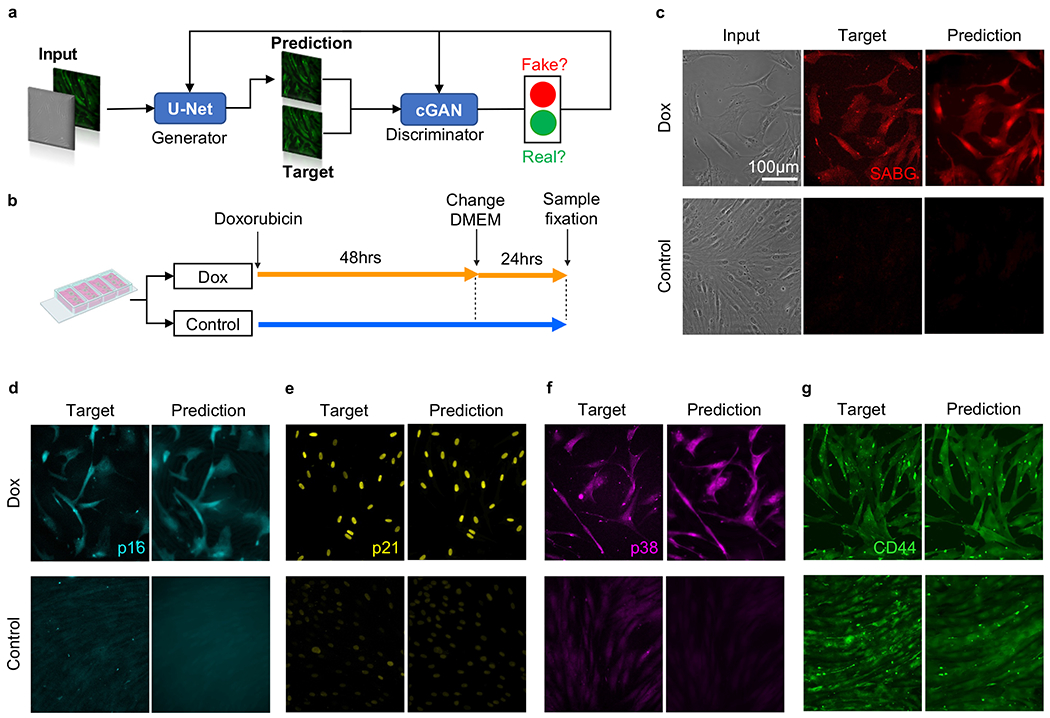
Doxorubicin-induced senescence marker prediction. (**a**) Machine learning model schematic. (**b**) Doxorubicin-treatment experimental timeline. Doxorubicin-treated (Dox) MSCs (orange) were cultured with Doxorubicin culture media for 48 hours followed by standard culture media for 24 hours prior to fixation. Control MSCs (blue) were cultured with standard culture media for 72 hours prior to fixation. (**c**) Fluorescent images and AI prediction for SABG. Left to Right: Phase-contrast images (Input), antibody-stained SABG immunofluorescence images (Target), and ML-produced SABG immunofluorescence images (Prediction). Top to Bottom: Doxorubicin-treated MSCs and Control MSCs. (**d-g**) Left to Right: Antibody-stained SABG immunofluorescence images (Target) and AI-produced SABG immunofluorescence images (Prediction). Top to Bottom: Doxorubicin-treated MSCs and Control MSCs. (**d**) p16. (**e**) p21. (**f**) p38. (**g**) CD44. Scale bar for **c-g** is 100 μm. (For interpretation of the references to colour in this figure legend, the reader is referred to the web version of this article.)

Two convolutional neural networks, a generator and a discriminator, form the AI model (Fig. 1a). The U-Net based generator(Ounkomol et al., 2018; Ronneberger et al., 2015) learns the relationship between a phase-contrast image and its corresponding immunofluorescent target image. During training the neural network quantifies the differences between the target and predicted image on the pixel level. The resulting prediction image of the generator is loaded into the discriminator network, which is a conditional generative adversarial network (cGAN)(Isola et al., 2017) and evaluates the pixel-to-pixel similarity of prediction and target image. Training is an iterative process, including a number of cycles through the model, which leads to optimization of the prediction. An individual training for each marker and each condition, treated and untreated, was completed, resulting in a trained model which was used to predict virtual fluorescent MSC senescent markers from phase-contrast images.

### Statistical analysis.

Data were reported as mean values ± standard deviation (SD). Statistical analysis was performed using Microsoft Excel and Python, and statistical significance was determined using 1-tailed paired t-tests. Hierarchical average clustering was performed using the ClustVis web tool. Different significance levels are indicated with asterisks in each figure caption. A p-value of less than 0.05 was considered statistically significant.

## 3. Results

In this work, we examined two types of senescence: stress-induced and replicative senescence. Both types of senescence share many features consistent with the anticipated hallmarks of senescence, including a decline in proliferation, morphological changes, and upregulated senescence marker expression (Debacq-Chainiaux et al., 2016; Turinetto et al., 2016; O’Hagan-Wong et al., 2016; Wagner et al., 2008; Weng et al., 2022).

To achieve stress-induced senescence, we tested two methods, namely Doxorubicin treatment and X-ray irradiation. To achieve replicative stress-induced senescence, we performed serial passaging of primary MSCs until passage 10 (Wagner et al., 2008; Liu et al., 2022; Estrada et al., 2013; Gu et al., 2016; Schellenberg et al., 2011). For all experiments, we utilized a U-net based conditional generative adversarial (cGAN) network for AI training (Fig. 1a). The AI model construction procedure and training are identical to that of our previous work(Imboden et al., 2021). As illustrated in Fig. 1a, we obtained phase contrast and immunofluorescent images of MSCs, and loaded them into the AI model for training. Upon training completion, we applied the AI model to new testing data and quantified the AI prediction accuracy by analyzing the Pearson correlation coefficient between the AI-predicted images and ground truth (i.e., actual immunofluorescent data). Using this approach, we investigated common senescence markers such as SABG(Lin et al., 2019; Oja et al., 2018), p16(Lin et al., 2019; Oja et al., 2018), p21(Oja et al., 2018), and p38(Thornton and Rincon, 2009) to detect senescent MSCs. Further details of the AI model development can be found in Materials and Methods.

### 3.1. AI-based phenotyping of stress-induced senescence

Our experiment and analysis of stress-induced senescence are not only important for evaluating cell culture quality, but are also physiologically relevant since continuous exposure to different stress types is often observed in native tissues(Debacq-Chainiaux et al., 2016). Multiple types of stress stimuli, which can be either chemical or physical, have been shown to cause DNA damage and subsequent cell cycle arrest (Debacq-Chainiaux et al., 2016; Özcan et al., 2016). To evaluate AI image translation labeling of senescent MSCs, we first examined two forms of stress-induced senescence, Doxorubicin-induced senescence and irradiation-induced senescence. We tested both of these stress-inducing agents to ensure the broad applicability of our AI method since previous studies have demonstrated that Doxorubicin and X-rays can produce their own unique morphological phenotype via distinct mechanisms (Özcan et al., 2016). Immortalized adipose-derived mesenchymcal stromal cells (adMSC) were utilized to obtain consistent samples and mitigate the effects of aging from cell passaging. For each tested senescence marker, we obtained at least 200 images for model training, in which we have shown that such an image number is sufficient to reach the maximal Pearson correlation coefficient between the target and AI prediction (i.e., maximal AI prediction accuracy)(Imboden et al., 2021).

#### 3.1.1. Doxorubicin-induced senescence

As demonstrated in Fig. 1b, we treated MSC samples with 0.5 *μ*M Doxorubicin for 48 hours(Kozhukharova et al., 2018), in which we tested three different dosages and identified that 0.5 *μ*M Doxorubicin generates a senescent response in MSCs without causing substantial cell death (Fig. S1). The MSCs were then incubated in base medium with the drug removed for 24-hours to allow for full expression of the senescent phenotype (Fig. S2) (Dezfouli et al., 2017). Following this, the samples were fixed and immunostained to later perform fluorescent imaging.

We show representative phase contrast images, immunostained images, and AI-predicted immunofluorescent images for the senescence markers senescence-associated beta-galactosidase (SABG), p16, p21, and p38 and the control marker CD44 in Figs. 1c–g. SABG is an eukaryotic hydrolase located in cellular lysosomes. After adjusting the pH value to 6 following fixation, SABG becomes detectable in senescent cells, but remains undetectable within young cells (Debacq-Chainiaux et al., 2016). SABG has been routinely used for a wide range of cell-based assays, and is thus currently the gold standard for senescence markers (Fig. 1c)(Li et al., 2017; Kozhukharova et al., 2018; Bashiri Dezfouli et al., 2020; Itahana et al., 2007; Lee et al., 2006; Li et al., 2017). P16 is a cell cycle arrest marker. High expression of p16 corresponds to inhibition of the S phase, indicative of cell cycle arrest (Fig. 1d). This S phase inhibition protects the cells from hyper-proliferation due to stress-induced DNA damage (Romagosa et al., 2011; Shimizu and Minamino, 2019; Liu et al., 2020). P21 is involved in transient cell cycle arrest as a response to acute DNA damage (Fig. 1e) (Shimizu and Minamino, 2019; Liu et al., 2020). P38 is a stress-activated mitogen-activated protein (MAP) kinase (Fig. 1f) (Shimizu and Minamino, 2019; Debacq-Chainiaux et al., 2016). Together, these markers effectively capture key hallmarks of senescence.

We found that all our selected senescence markers exhibited upregulated expression levels in Doxorubicin-treated cells (Figs. 1c–g Target) where such upregulated expression levels were accurately predicted by the AI model (Figs. 1c–g Prediction). In addition, the AI model was able to capture intercellular heterogeneity of marker expression level. As demonstrated in Figs. 1c and d, there is an apparent cell-level variation of SABG and p16 expression, in which the target and prediction exhibit highly similar intensity distributions. The cell morphology (Figs. 1c and d, sub-cellular structures such as the nuclear shape (Fig. 1e) were also captured in the prediction images. CD44 was utilized as a control marker to validate the AI prediction specificity. CD44 is a MSC surface marker that is expressed regardless of the senescence state(Voga et al., 2021). As anticipated, there were negligible intensity differences in CD44 expression between the treated and untreated MSC target and prediction samples indicating proper staining of samples (Fig. 1g).

**Fig. 2.**
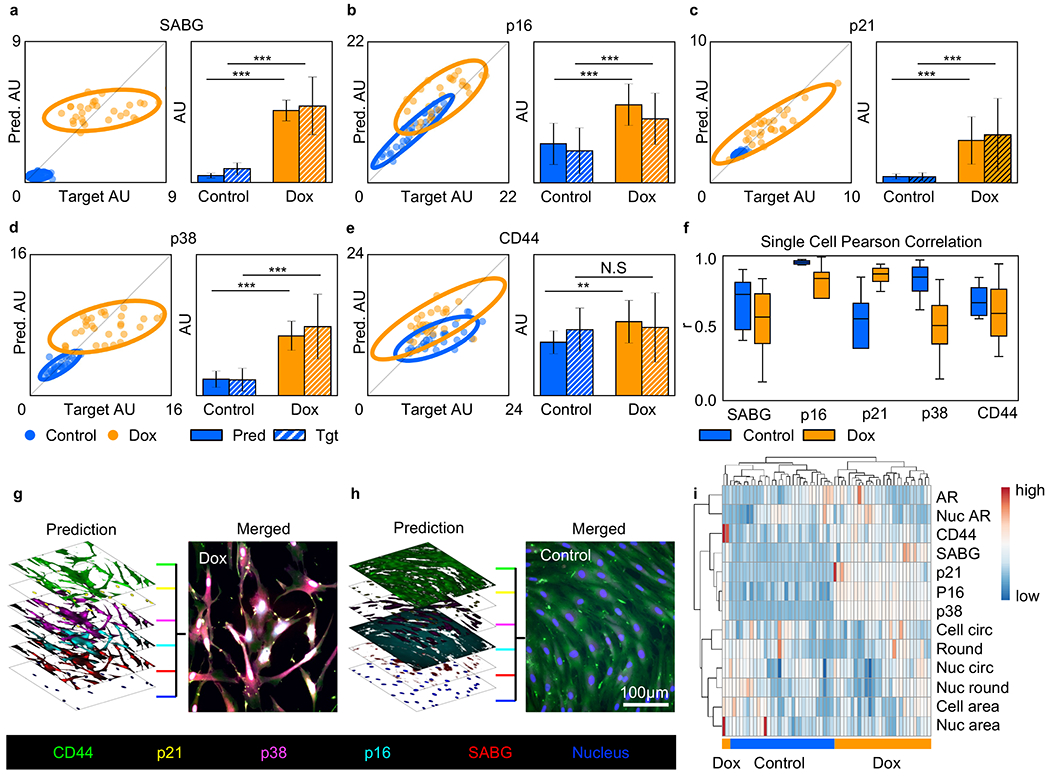
Doxorubicin-induced senescence marker quantification. (**a-e**) Doxorubicin-induced senescence scatter plots and bar charts. (**a**) SABG. (**b**) p16. (**c**) p21. (**d**) p38. (**e**) CD44. Scatter plots demonstrate a strong positive correlation between target and prediction images for control MSCs and Doxorubicin-treated MSCs stained for p16, p21, and CD44. Scatter plots demonstrate a moderate positive correlation between target and prediction images for control MSCs and Doxorubicin-treated MSCs stained for SABG and p38. Bar charts demonstrate a corresponding significant difference in SABG, p16, p21, and p38 between control-MSCs and Doxorubicin-treated MSCs for both prediction and target. Bar charts demonstrate a significant difference and no significance in CD44 between control-MSCs and Doxorubicin-treated MSCs for prediction and target, respectively. The gray diagonal lines denote a perfect prediction-target correlation. N.S. not significant; *p < 0.05; **p < 0.001; ***p < 0.0001. (**f**) Single cell Pearson correlation between prediction and target iamges for Doxorubicin-treated MSCs stained for SABG, p16, p21, p38, and CD44. Each Pearson correlation box plot contains 30 target-prediction image pairs. All markers demonstrate a moderate to strong Pearson correlation coefficient (r) greater than 0.5. Tgt: Target, Pred: Prediction (**g, h**) Multi-marker composite images demonstrating CD44, p21, p38, p16, SABG, and nucleus predictions from single phase-contrast images. (**g**) Doxorubicin-treated. (**h**) Control group. Scale bar is 100 μm for **g** and **h**. (**i**) Doxorubicin-treated MSC characteristic heatmap. Outlined cells were hierarchically clustered according to marker intensity and eight morphological features leading to clear clustering patterns. AR: Aspect-ratio; Nuc: Nucleus; Circ: Circularity; Round: Roundness.

To quantify the AI prediction accuracy, the mean fluorescent intensity for each marker was measured in 30 target and prediction single cells. The target-prediction correlation was analyzed by obtaining the Pearson-correlation coefficient of the scatter plots as shown in Figs. 2a–e). Each point represents one manually-outlined cell contour. As indicated by the 95% confidence ellipses, we observed distinct clustering of treated (orange) and control (blue) cells for all tested senescence markers. This finding was further confirmed by the corresponding intensity bar chart plots, as we found that the AI-predicted senescence marker intensities were significantly higher than the control intensities. We also found consistent CD44 expression between the target and prediction (Fig. 2e), as anticipated. We, however, noted differential CD44 expression between the treated and control samples for the AI prediction, which may be mitigated with further training and measurement sampling. To confirm the robustness of AI labeling, we repeated the experiment and lowered the seeding density of the control sample, effectively compensating for the cell loss due to treatment (Fig. S3). We examined the p38 expression level and observed agreement between the target and prediction for both the test images reserved from the training set (Fig. S3b) and images of an independent biological replicate (Figs. S3c and S3d). Here, the independent sample was cultured, fixed, stained, and imaged separately from the samples used for AI training. These results collectively validated our U-Net+cGAN model’s ability to label senescent adMSCs.

In addition, the scatter plots for p16, p21, p38, and CD44 indicate a positive target-prediction correlation within each sample. This correlation suggests that the cell-level marker expression heterogeneity, which was illustrated by the spread of the data points, was appropriately captured by AI. We note that such intrapopulation heterogeneity, however, was not clearly observed in SABG (Fig. 2a). This finding suggests that SABG expression might not be strongly associated with the morphological phenotype within individual populations. We further summarized the target-prediction correlation for all markers by plotting the marker’s Pearson correlation coefficients in Fig. 2f. Overall, our AI model effectively identified marker expression of senescent MSCs and captured the intra-population cell heterogeneity best for p16 (r ~ 0.8) and p21 (r ~ 0.7). Further marker prediction analysis is represented in bivariate plots (Fig. S4), a principal component analysis (PCA) biplot (Fig. S5a), and uniform manifold approximation and projection for dimension reduction (UMAP) (Fig. S5b) which again confirm the clear separation between Doxorubicin-treated and untreated samples. Lastly, quantification of the pixel-pixel Pearson correlation coefficient across the entire field-of-view demonstrated moderate correlations for all markers (Fig. S6). The pixel-level correlation coefficient is dependent on the signal-to-noise (SNR) ratio of target images used for AI training, consistent with previous findings (Imboden et al., 2021). Our demonstrated Pearson-signal-to-noise ratio shows a positive correlation for the Doxorubicin-treated group and a negative correlation for untreated group (Fig. S7).

In contrast to traditional immunofluorescence microscopy that typically requires intensive sample preparations for multiplex measurements, our AI labeling method allowed us to directly combine multiple AI-predicted marker expression measurements with minimal sample preparation time. This capability was illustrated by the 6-color composites shown in Figs. 2g and h. The expression of all senescence markers was observed in the Dox-treated cells (Fig. 2g) whereas only CD44 is expressed in the control sample (Fig. 2h). Multi channel composite capabilities similarly allowed us to perform a multi-component analysis (Fig. 2h). Hierarchical clustering heatmap showed a clear segregation of Dox-treated and untreated cells (Fig. 2i). Furthermore, the morphological phenotype and senescence marker expression provided complementary characterizations of MSCs. This suggests that combining both morphology-based and molecular-based measurements may better describe the heterogeneity of cell state and function.

#### 3.1.2. Irradiation-induced senescence

**Fig. 3.**
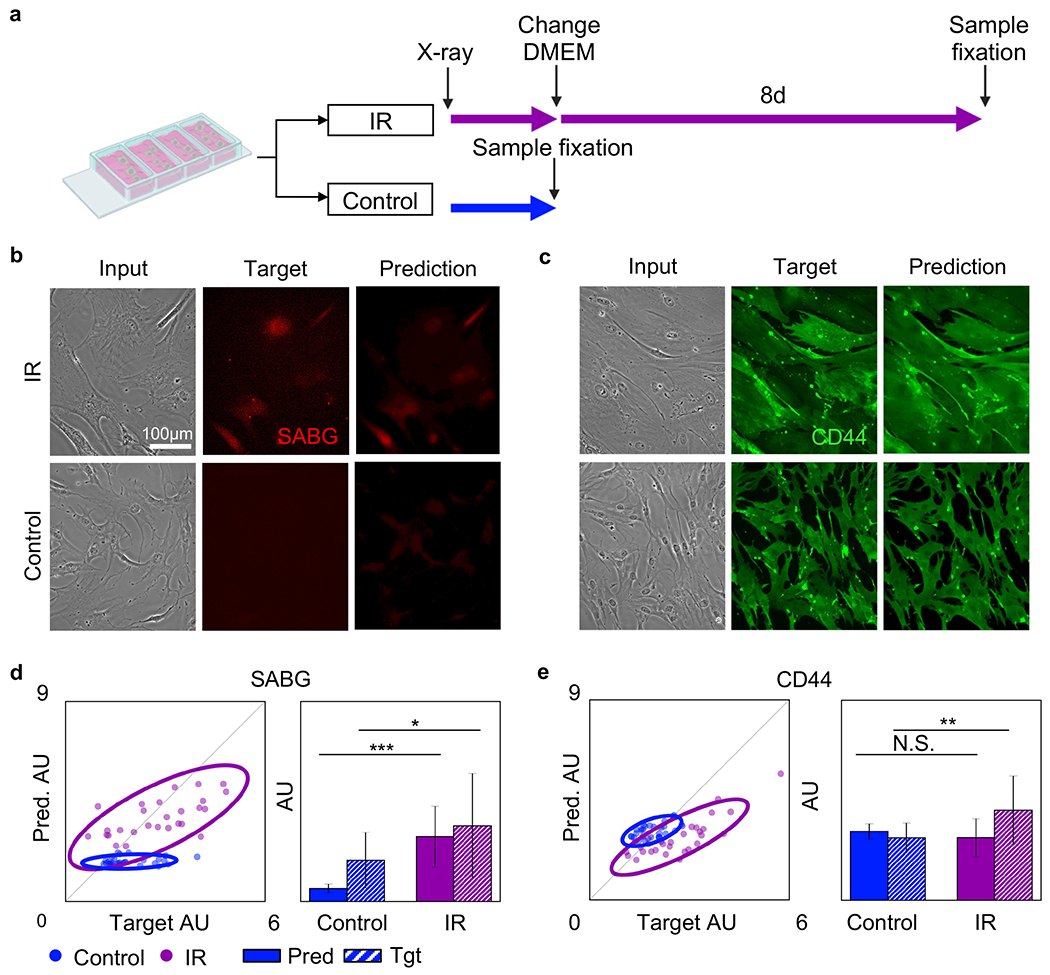
Irradiation-induced senescence marker prediction and quantification. (**a**) Irradiation-treatment experimental timeline. Irradiation-treated MSCs (purple) were exposed to irradiation followed by culture in standard culture media for 8 days prior to fixation. Control MSCs (blue) were cultured in standard culture media for 8 days prior to fixation. (**b, c**) Left to Right: Phase-contrast images (Input), antibody-stained immunofluorescence images (Target), and AI-produced immunofluorescence images (Prediction). Top to Bottom: Irradiation-treated MSCs and Control MSCs. (**b**) SABG. (**c**) CD44. Scale bar is 100 μm for **b** and **c**. (**d, e**) Irradiation-induced senescence scatter plots and bar charts. (**d**) SABG. (**e**) CD44. Scatter plots demonstrate a strong positive correlation between target and prediction images for SABG IR, CD44 Control, and CD44 IR. Scatter plots demonstrate a weak correlation between target and prediction images for SABG Control. Bar charts demonstrate a corresponding significant difference in SABG between control-MSCs and irradiation-treated MSCs for prediction and target. Bar charts demonstrate no significance and a significant difference in CD44 between control-MSCs and irradiation-treated MSCs for prediction and target, respectively. N.S. not significant; * p < 0.05; ** p < 0.001; *** p < 0.0001. Tgt: Target, Pred: Prediction.

We further explored whether the irradiation-induced senescence can be appropriately labled using our U-Net+cGAN model. The radiation oncology application of MSC therapy has received much attention in the past decade (Wang et al., 2021; Maria et al., 2016;Hmadcha et al., 2020; Zhou et al., 2021). The MSC X-ray irradiation (IR) responses, including proliferation, differentiation potential, and immunomoduluation capacity, directly determine the therapeutic efficacy. In this experiment, we primarily study the gold standard senescence marker, SABG, for simplicity. The experimental timeline is summarized in Fig. 3a. In brief, after the IR treatment, the cell culture medium was refreshed and samples were cultured for 8 additional days to observe a morphological difference between irradiated and control samples. For example, the irradiated MSCs exhibit a slightly larger cell area compared to the untreated cells (Fig. S8a), as shown by the phase contrast images in Fig. 3b. The control sample was untreated and fixed on the IR treatment day to prevent cell over-growth. We found that a 200 Gy dosage induced morphological changes while maintaining a high cell viability ~ 95% (Fig. S8b). Specifically, we treated cells with a dose rate of 8.5 Gy/min for 23 minutes and 32 seconds. The high IR dosage requirement may result from the overexpression of telomerase reverse transcriptase in immortalized adMSCs. Phase-contrast, SABG immunofluorescence, and AI-predicted SABG immunofluorescence images of adMSCs are displayed in Fig. 3b. The IR-induced upregulation of SABG expression is observed in both immunofluorescence target and AI-predicted images for the irradiated samples. This indicates that our AI model correctly identified senescent MSCs marker expression. CD44 (Fig. 3c) showed a similar expression level in treated and control samples for both target and AI prediction, consistent with our anticipated results and previous findings. Like the Doxorubicin analysis, the AI prediction performance was evaluated by measuring the signal intensity for individual cells. The mean signal of 30 outlined single cells from target and prediction images for SABG (Fig. 3d) and CD44 (Fig. 3e) was analyzed. The 95% confidence ellipses indicated a clear separation between IR-treated (purple) and untreated (blue) populations. This distinct clustering and the ~45° data trend collectively indicate that both actual fluorescent (target) and AI-predicted data can be used for estimating the IR-upregulated SABG expression. The mean intensity value for both populations was calculated and the results were displayed through bar chart plots (right figure of Fig. 3d). The AI-predicted SABG intensity of IR-treated MSCs was significantly higher than that of control MSCs, confirming the AI’s ability to identify senescent MSCs. The pixel-wise target-prediction correlation for SABG and CD44 was summarized by plotting the marker’s Pearson correlation coefficients in Fig. S9. The bivariate plot (Fig. S10), PCA biplot (Fig. S11a) and UMAP (Fig. S11b) collectively confirmed the clear AI-predicted separation between the irradiated and control groups.

### 3.2. AI-based phenotyping of replicative senescence

**Fig. 4.**
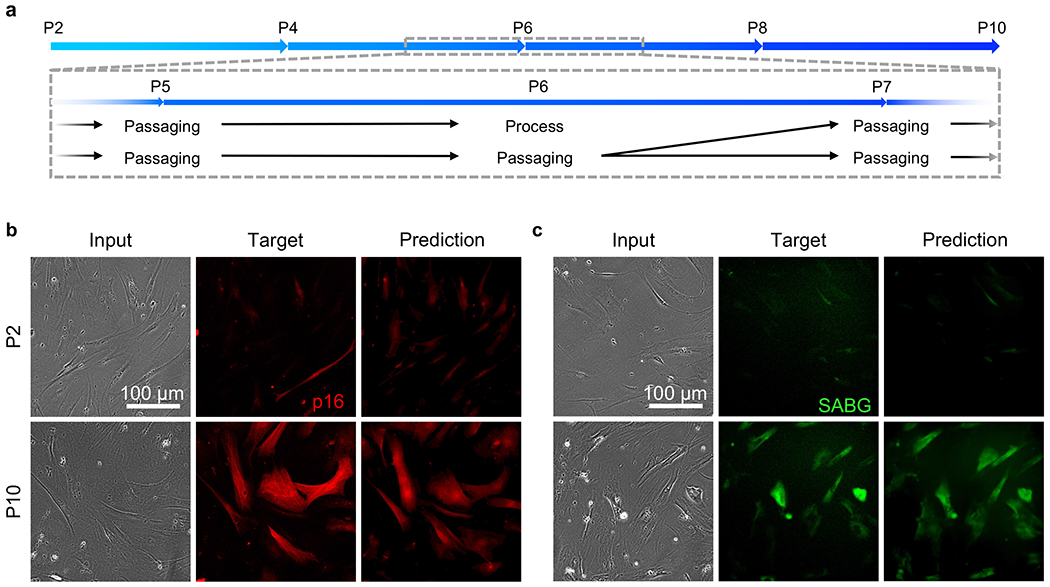
Replicative senescence marker prediction. (**a**) Replicative senescence experimental timeline. MSCs were cultured in standard 6-well culture plates (6 Well) using either FBS-supplemented DMEM or StemFit MSC culture media for 9 passages, passage 2 to passage 10. Every even passage (e.g. P2, P4, etc.), one 6 well plate of MSCs was fixed. (**b, c**) Left to Right: Phase-contrast images (Input), antibody-stained immunofluorescence images (Target), and AI-produced immunofluorescence images (Prediction). Top to Bottom: Passage 2 MSCs and passage 10 MSCs. (**b**) p16. (**c**) SABG. Scale bar 100 μm.

After confirming the accuracy of our AI model in predicting senescence in MSCs that have undergone stress-induced senescence via two distinct mechanisms, we studied the performance of our proposed AI platform in characterizing replicative senescence. Testing whether our model can be applied in such a setting is critical, since *in vitro* expansion of MSCs is known to inevitably lead to cellular aging, in which telomeres are shortened during each cell division and autophagy dysregulation arises over time(Beausejour, 2007; Yang et al., 2018). The experimental timeline for the generating replicative senescence MSCs is summarized in Fig. 4a. In brief, we cultured primary bone marrow-derived MSCs (bmMSCs) over 10 passages, in which half of the MSC samples were fixed every other passage. Here, the use of primary cells, rather than immortalized cell lines, allows us to model the slowdown of cell proliferation, and hence replicative senescence, as shown by the plateaued cell growth curve in Fig. S12. To ensure our AI tool’s wide applicability, we tested two media conditions, 10% FBS-supplemented DMEM and serum-free StemFit MSC media. For simplicity, we presented the FBS-DMEM result in the main manuscript and included the StemFit MSC data in SI (Figs. S13 and S14 for images and quantification, respectively). The replicative senescence state of passage-10 (P10) MSCs was confirmed by the plateaued proliferation curves (Fig. S12), which indicate cell cycle arrest. We showed representative target immunofluorescence images of senescence markers p16 (Fig. 4b) and SABG (Fig. 4c), and confirmed that both markers were significantly upregulated in P10 MSCs. We further found that such upregulated expression levels were accurately predicted by our AI model. In this experiment, CD105, a standard bmMSC surface marker, was used as a control marker and found to be relatively constant from P4 to P10 (Fig. S14).

**Fig. 5.**
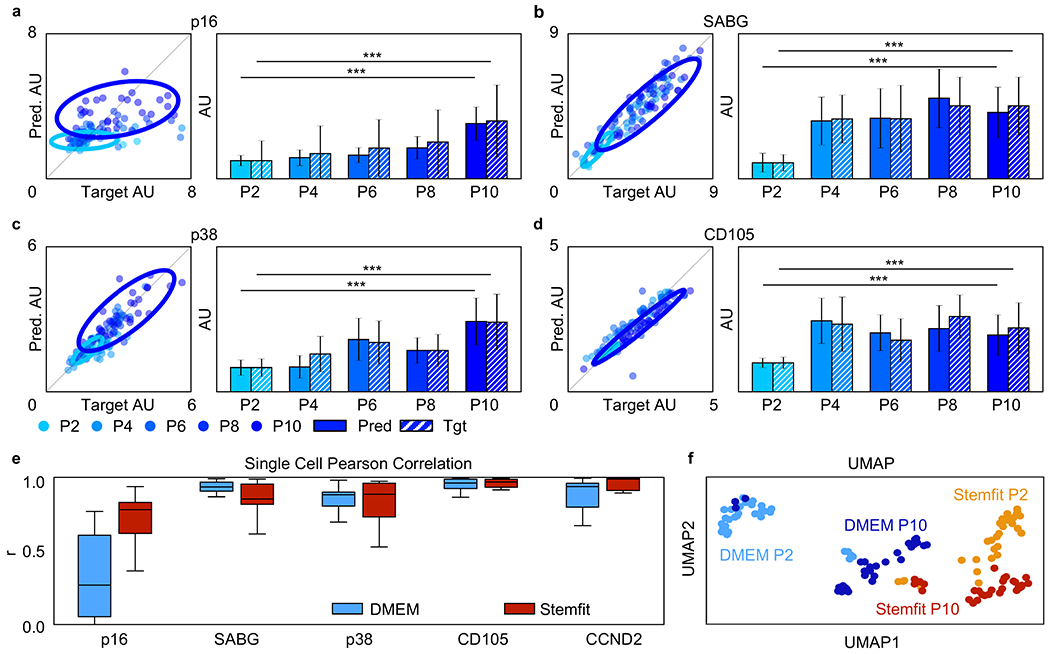
Replicative senescence marker quantification. (**a-d**) Replicative senescence scatter plots and bar charts. (**a**) p16. (**b**) SABG. (**c**) p38. (**d**) CD105. Scatter plots demonstrate a strong positive correlation between target and prediction images for passage 2 MSCs to passage 10 MSCs stained for p16, SABG, p38, and CD105. Bar charts demonstrate a corresponding significant difference in p16, SABG, p38, and CD105 between passage 2 MSCs and passage 10 MSCs for prediction and target. N.S. not significant; *p < 0.05; **p < 0.001; *** p < 0.0001. Tgt: Target, Pred: Prediction. Scatter plots and bar charts were created using Python version 3.7. (**e**) Single cell Pearson correlation for replicative senescence MSCs stained for p16, SABG, p38, CD105, and CCND2. Here, CCND2 was also tested since it has been commonly found to be upregulated in senescent MSCs (Bertolo et al., 2019). Each Pearson correlation box plot contains 30 target-prediction image pairs. All markers demonstrate a moderate to strong Pearson correlation coefficient (r) with r values greater than 0.5. (**f**) Replicative senescence MSC UMAP. UMAP demonstrates separation of passage 2 MSCs from passage 10 MSCs and MSCs cultured in DMEM culture media from MSCs cultured in Stemfit culture media.

To quantify the AI prediction accuracy, we measured the mean fluorescent intensity of each marker (i.e., p16, SABG, p38, and CD105) for 30 cells for each tested passage. We then plotted both the target and AI-predicted values in Figs. 5a–d. As shown by the scatter plots, a segregation of young cells represented by P2 (light blue) and old cells represented by P10 (dark blue) can be observed by the 95% confidence ellipses for p16 (Fig. 5a), SABG (Fig. 5Fig. 5c), whereas no clear segregation was observed for CD105 (Fig. 5d). We noted a jump in CD105 expression between P2 and P4. This jump may be related to the cell recovery from cryopreservation(Davies et al., 2014). As illustrated by the bar charts, we found that both the target (solid-fill area) and prediction (striped area) show very similar marker expression trends for all tested markers. We further performed t-test for all passage combinations for the FBS media condition and observed similar p-value distributions for both prediction and target (Fig. S15).

We also found that the AI model can capture the cell-level variation of marker expression level for SABG, p38, and CD105, as indicated by the positive correlation between target and prediction values. Such correlation is summarized by their corresponding Pearson correlation coefficients in Fig. 5e. Together, these results suggest that our AI platform can accurately identify replication-induced senescent MSCs resulting from *in vitro* expansion. Taking advantage of our AI method’s multiplex capability, we performed a UMAP analysis by combining all 6 marker measurements. As illustrated in the UMAP plot (Fig. 5f), we observed four distinguishable populations that correspond to the P2 and P10 MSCs in DMEM and StemFit cultures. We further found that the UMAP distance between P2 and P10 for the StemFit culture is shorter than that for the DMEM culture. This finding suggests that the use of StemFit medium might mitigate the upregulation of senescence marker expression during *in vitro* expansion. Such findings were further confirmed by bivariate (Fig. S16) and PCA (Fig. S17) biplots.

## 4. Discussion and conclusion

Mitigating the effects of *in vitro* cell senescence has been a major challenge in generating MSCs that can achieve effective and reproducible therapy outcomes (Sharma et al., 2014; Zhao et al., 2015). Thus, it is critical to develop fast, label-free, and robust cell characterization methods for quality control in MSC manufacturing(Bertolo et al., 2019). In this work, we reported a microscopy-based method that can characterize stress-induced senescence and replicative senescence in MSCs *in situ*. We showed that our AI algorithm can accurately predict senescence marker expression induced by Doxorubicin, X-ray irradiation, and long-term culture. We observed increased marker signals in the AI-predicted fluorescent images for multiple senescence markers (e.g., SABG, p16, p21, and p38). Quantitative assessments of the AI prediction accuracy indicated a strong positive prediction-target correlation for most tested senescence markers.

As a demonstration, this work focused on an adMSC line and primary bmMSCs from one donor. To further examine the generalizability of our method, it is valuable to test other MSC sources in the future. For example, it would be interesting to investigate how the donor-to-donor variability impacts the performance of our AI method. Testing MSCs derived from induced pluripotent stem cells (iPSC-MSCs), which have been used in refractory graft-versus-host-disease (GVHD) in clinical trials is also important(Lian et al., 2016; Bloor et al., 2020). Beside the cell source, the effects of experimental conditions, such as senescence-inducing agents, seeding density, and imaging systems on the AI prediction should also be investigated in the future. We also note that a few limitations of our AI approach remain to be addressed in future work. First, our method’s ability to label subcellular structures, such as ultrastructures of organelles and vesicles, remains to be confirmed. Second, we found that the cell-level AI prediction accuracy is suboptimal when the training dataset (i.e., immunofluorescence images) has a low signal-to-noise ratio. This issue can be particularly seen in the SABG (Fig. 3) and p16 data (Fig. 4) in the irradiation and replicative senescence experiments, respectively.

While we have obtained proof-of-concept results and demonstrated the utility of our AI-based MSC phenotyping method, additional technical challenges need to be addressed before it can be routinely implemented in cell manufacturing. To improve the AI training outcome, it is imperative to further optimize the staining and imaging procedures and incorporate new AI training frameworks, such as transfer learning and data augmentation techniques. In addition, the markers tested in this work are not strictly functional markers that report the immunomodulation capacity or differentiation potential. In future work, it would be useful to investigate how our AI model can predict expression of other previously studied markers, including telomere associated protein Rap1(Poon et al., 2015) , mitochondrial reactive oxygen species(Li et al., 2019) , mitochondrial morphology(Zhang et al., 2020) , and senescence-associated secretory phenotype(Alessio et al., 2019). Further, it would be useful to investigate whether deep-learning models can directly predict MSC functions based on transmitted light microscopy. Overall, our AI approach readily provides many advantages over current immunochemistry-based methods. Our platform can be easily adapted to different cell sources (e.g., adipose-derived or bone-marrow-derived MSCs), allowing us to account for the population heterogeneity that arises from the tissue origin and donor-donor variability. Our AI-labeling tool offers a simple way to perform multi-marker cell characterizations, as illustrated in Figs. 2 g–i and Fig. 5f. The AI training can include as many markers as necessary, enabling a cost-effective way to obtain combinatorial descriptions of the MSC state. Such combinatorial descriptions can be useful for deciphering the activities of different senescent pathways and better utilizing surface markers that are not exclusively expressed in senescent cells. Lastly, the simultaneous molecule-based measurements and morphological characterization can be used to study the relationship between senescence pathways and morphological phenotypes. When combining with other noninvasive phenotyping methods, such as autofluorescence measurements(Bertolo et al., 2019) and multiplex measurements of senescence-associated secretory phenotype (Alessio et al., 2019), our AI tool may provide additional information to further characterize the senescence state. These advantages collectively make our platform a useful tool to better understand MSC senescence and develop corresponding mitigation strategies.

## Supplementary Material

SI

## Data Availability

Accession codes Software for training and an example dataset is available at https://xuanqing94.github.io/ai-reporter/. The senescence data for this work are stored in https://github.com/bleeve007/AI_Prediction_MSC_Senescence.git.

## Data availability

Data will be made available on request.
