# Supplementary material for: Phenotyping senescent mesenchymal stromal cells using AI image translation
Source: Curr Res Biotechnol. Author manuscript; Available in PMC 2023 Dec 1. (PMC10691861; doi:10.1016/j.crbiot.2023.100120)
Supplement: SI [file NIHMS1900554-supplement-SI.pdf]

Supplementary Information for

Phenotyping Senescent Mesenchymal Stromal Cells using AI Image Translation

Leya Weber[1],+, Brandon S. Lee[2],+, Sara Imboden[1], Cho-Jui Hsieh[3], and Neil Y. C. Lin[1,2,4]*

This pdf file includes:
Figs. S1 through S17

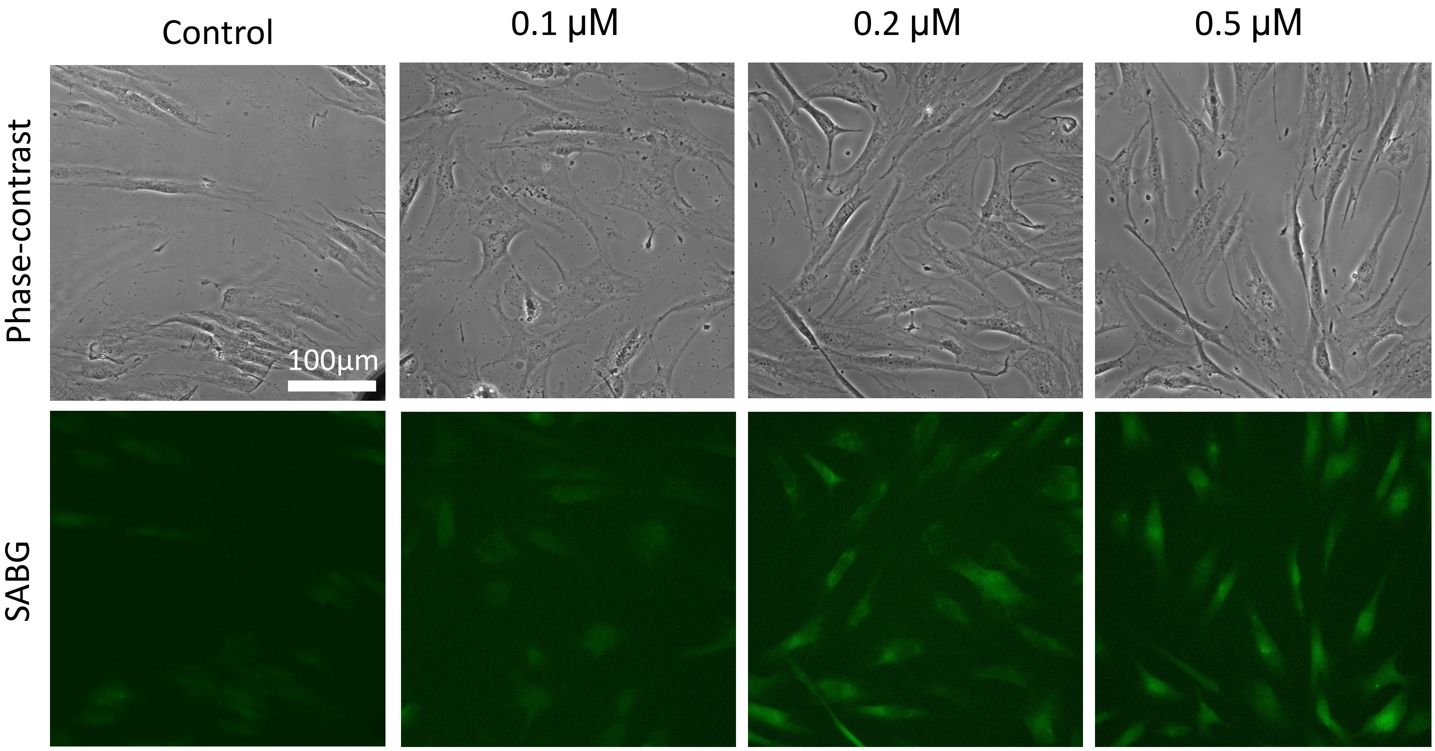

Figue S1. Doxorubicin-treated adMSCs screening assay. Top to Bottom: Phase-contrast images (Input) and SABG-stained immunofluorescence images (Target). Cells were treated with various concentrations of Doxorubicin to induce senescence. 0.5 μM Doxorubicin was used in experiments to induce senescence as it resulted in intense expression of SABG without significantly altering cell health or morphology. Higher concentrations tested (not shown) induced cell death.

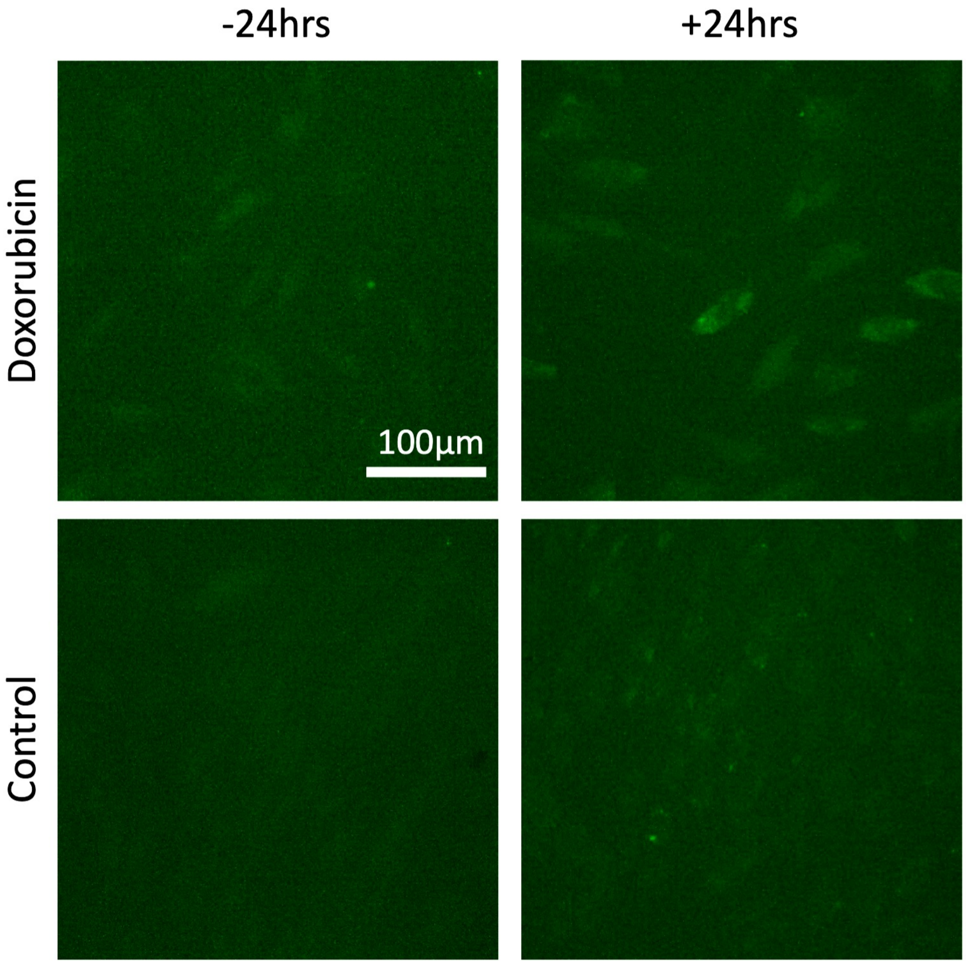

Figure S2. adMSC SABG expression of Doxorubicin-treated adMSCs. Left to Right: MSCs without (-) and with (+) 24 hours recovery time. Top to Bottom: 0.1µM Doxorubicin-treated MSCs and Control MSCs. Following the 48-hour Doxorubicin treatment, the media containing Doxorubicin was removed and replenished with base medium (without drug) for 24-hours to allow for full expression of the senescence phenotype.

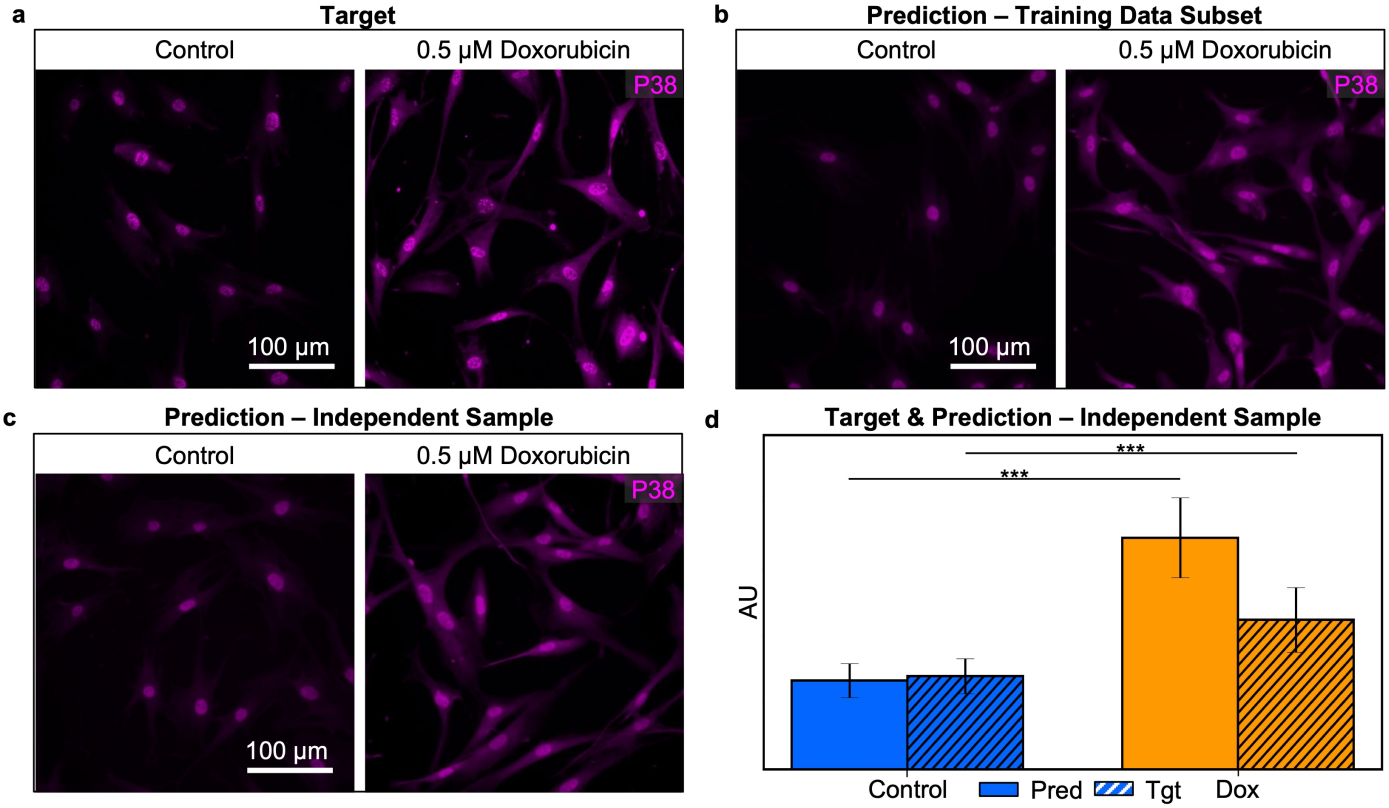

Figure S3. Validation of AI predictions of p38 expression level. (**a**) Representative target images in the training dataset. (**b**) Predicted fluorescent images obtained from a subset of phase contrast test images that were reserved from training. (**c**) Predicted fluorescent images obtained from phase contrast images of biologically independent samples. The staining and imaging of this sample were performed independently to that of samples used for AI training. (**d**) Single cells analysis (15 cells per condition) based on the data shown in **(c)** confirms the AI's ability to distinguish control and treated cells. N.S. not significant; * $p < 0.05$; ** $p < 0.001$; *** $p < 0.0001$.

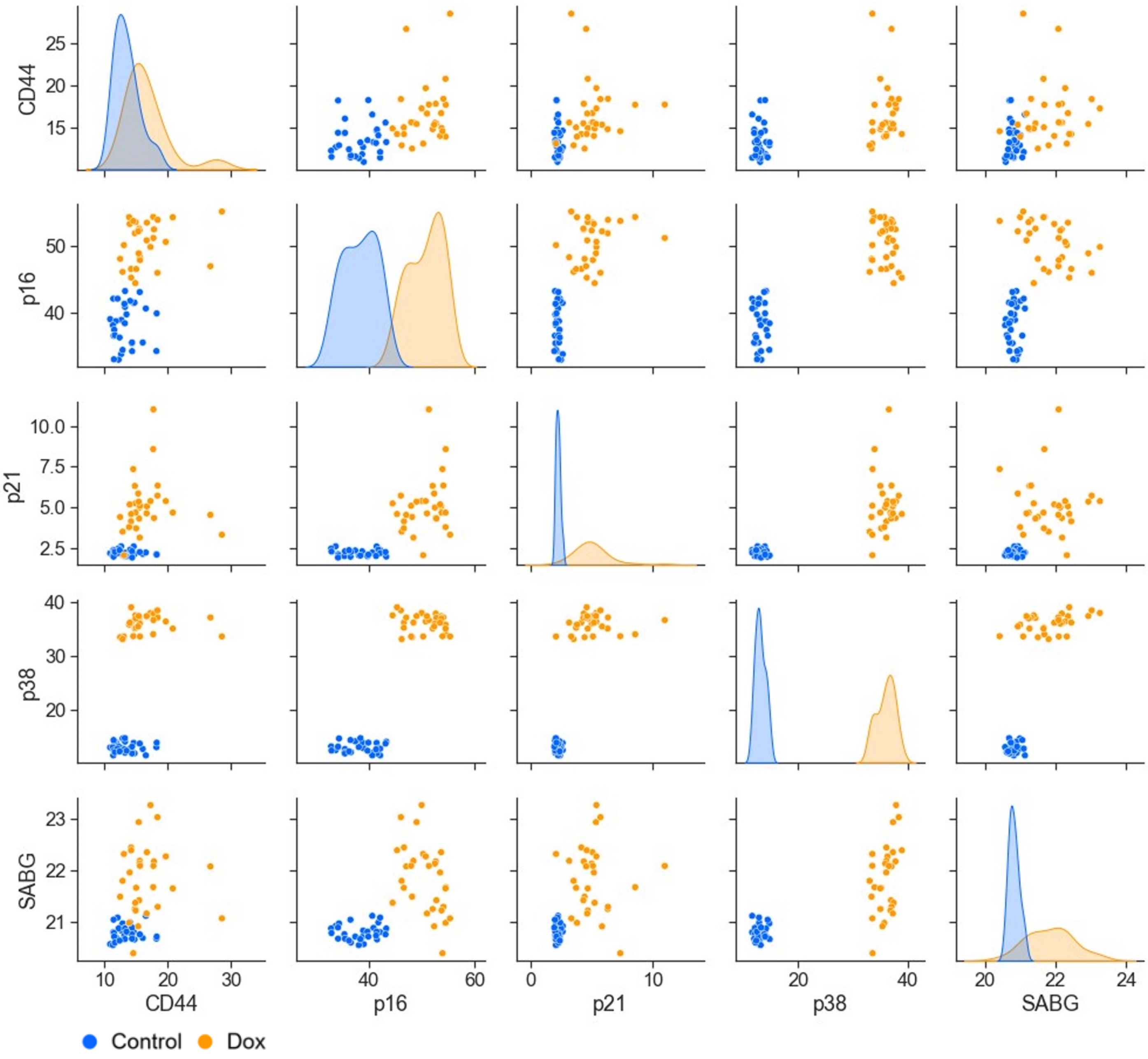

Figue S4. Doxorubicin bivariate plots. Several phase contrast images for control MSCs and Doxorubicin-treated (Dox) MSCs were pooled for analysis. Representative phase contrast images are shown in Fig. 1c. Images were inputted into the AI model for marker expression prediction. MSCs in predicted images were individually outlined. Bivariate plot represents the AI model predicted expression of CD44, p16, p21, p38, and SABG in 30 control MSCs and 30 Dox MSCs. In these scatter plots, one data point represents one cell. Histograms demonstrate intensity distributions for each respective condition. These results confirm the distinct separation between Doxorubicin-treated and untreated samples, also illustrating the Doxorubicin's effectiveness in inducing senescence.

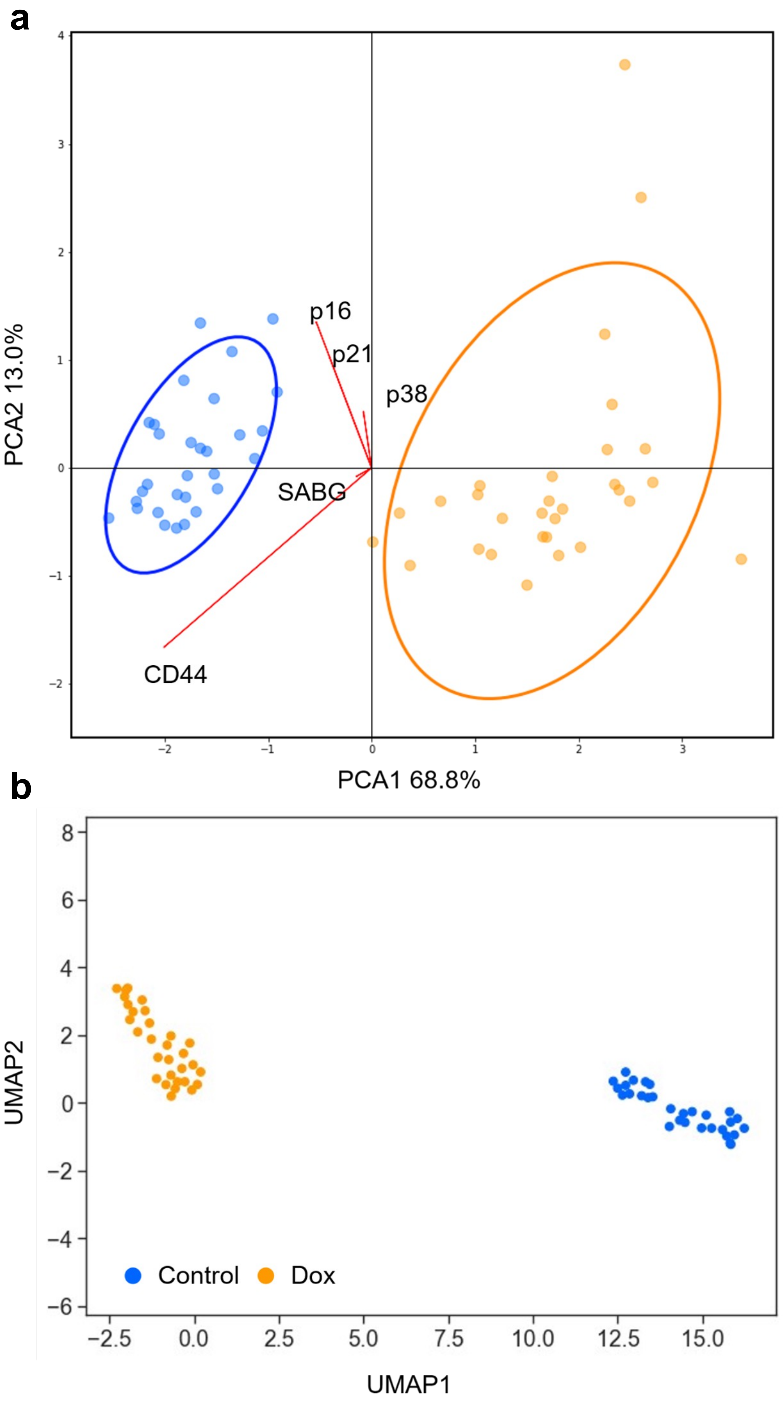

Figue S5. Doxorubicin PCA biplot and UMAP. Several phase contrast images for control MSCs and Doxorubicin-treated (Dox) MSCs were pooled for analysis. Representative phase contrast images are shown in Fig. 1c. Images were inputted into our AI model for marker expression prediction. MSCs in predicted images were individually outlined. PCA biplot (**a**) and UMAP (**b**) represent the AI model predicted expression of CD44, p16, p21, p38, and SABG in 30 control MSCs and 30 Dox MSCs. 95% confidence ellipse is shown in PCA biplot. PCA biplot and UMAP demonstrate a clear distinction between control MSCs and Dox MSCs.

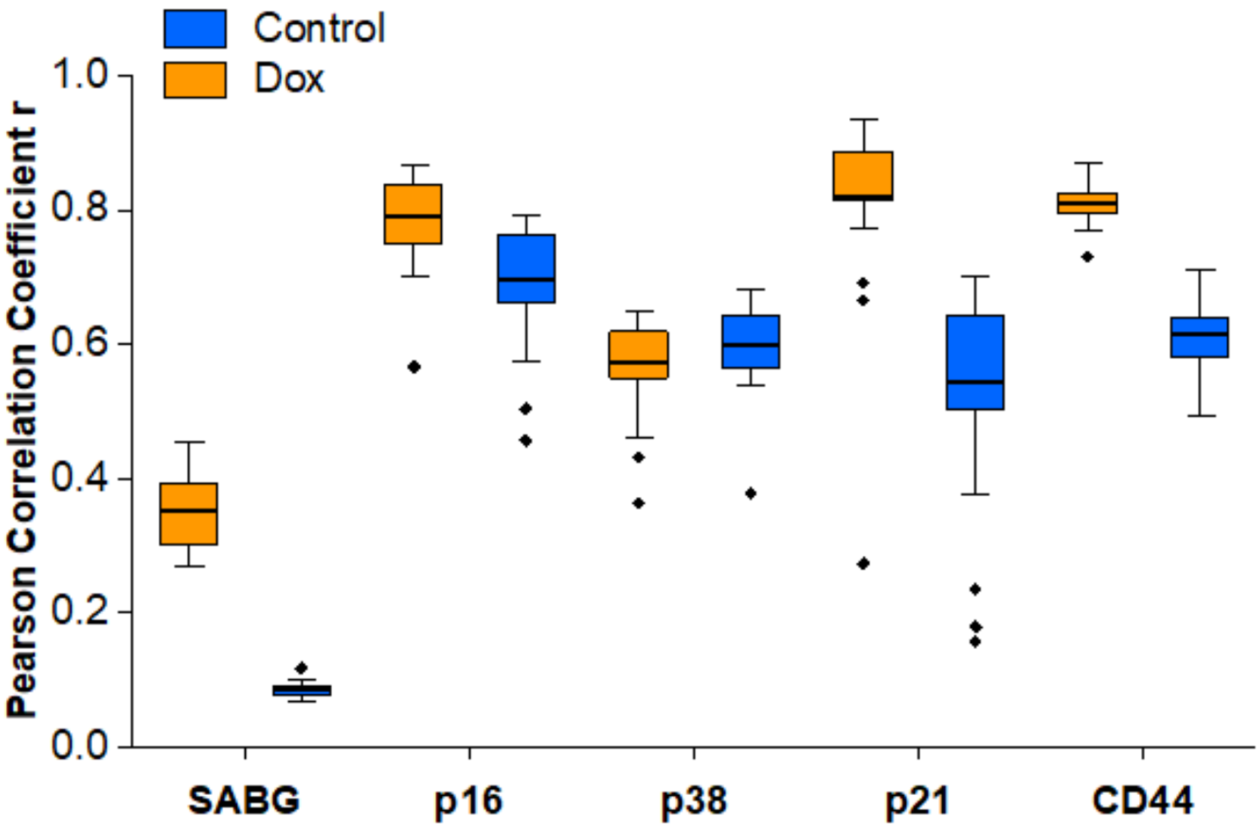

Figue S6. Pixel-wise Pearson correlation coefficient between target and prediction images of immunofluorescence images of Doxorubicin-treated adMSCs. Each box represents 26 phase contrast images that were pooled for analysis. Representative phase contrast images are shown in Fig. 1c. Data was obtained by comparing every pixel occupying the entire FOV of the prediction image to that of the target for both control images and Doxorubicin-treated adMSCs. Results suggest a moderate correlation for all considered markers.

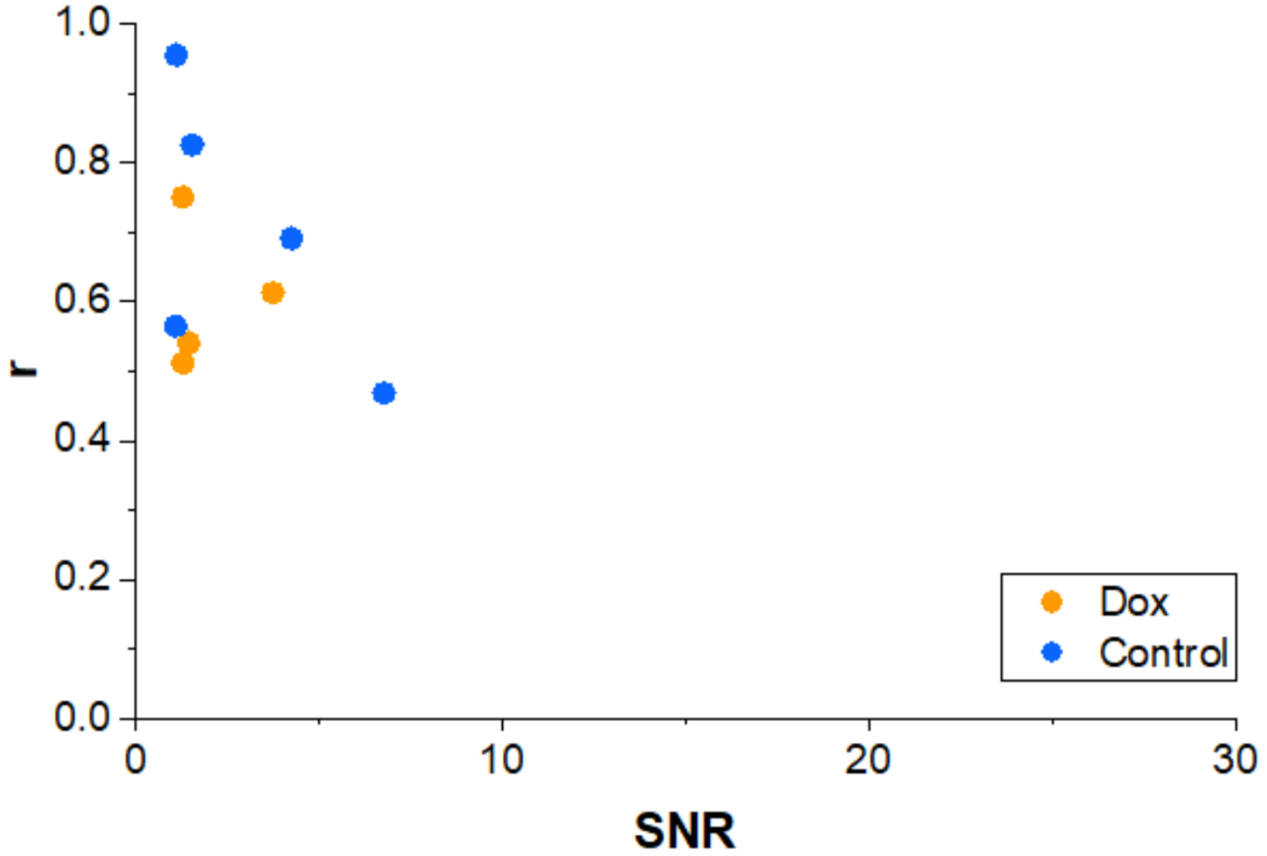

Figue S7. Pearson signal-to-noise ratio (SNR) of Doxorubicin-treated adMSCs. Several phase contrast images for control MSCs and Doxorubicin-treated (Dox) MSCs were pooled for analysis. Representative phase contrast images are shown in Fig. 1c. A negative correlation between Pearson value and Signal-to-Noise is demonstrated for control group and a positive correlation for Doxorubicin-treated MSCs.

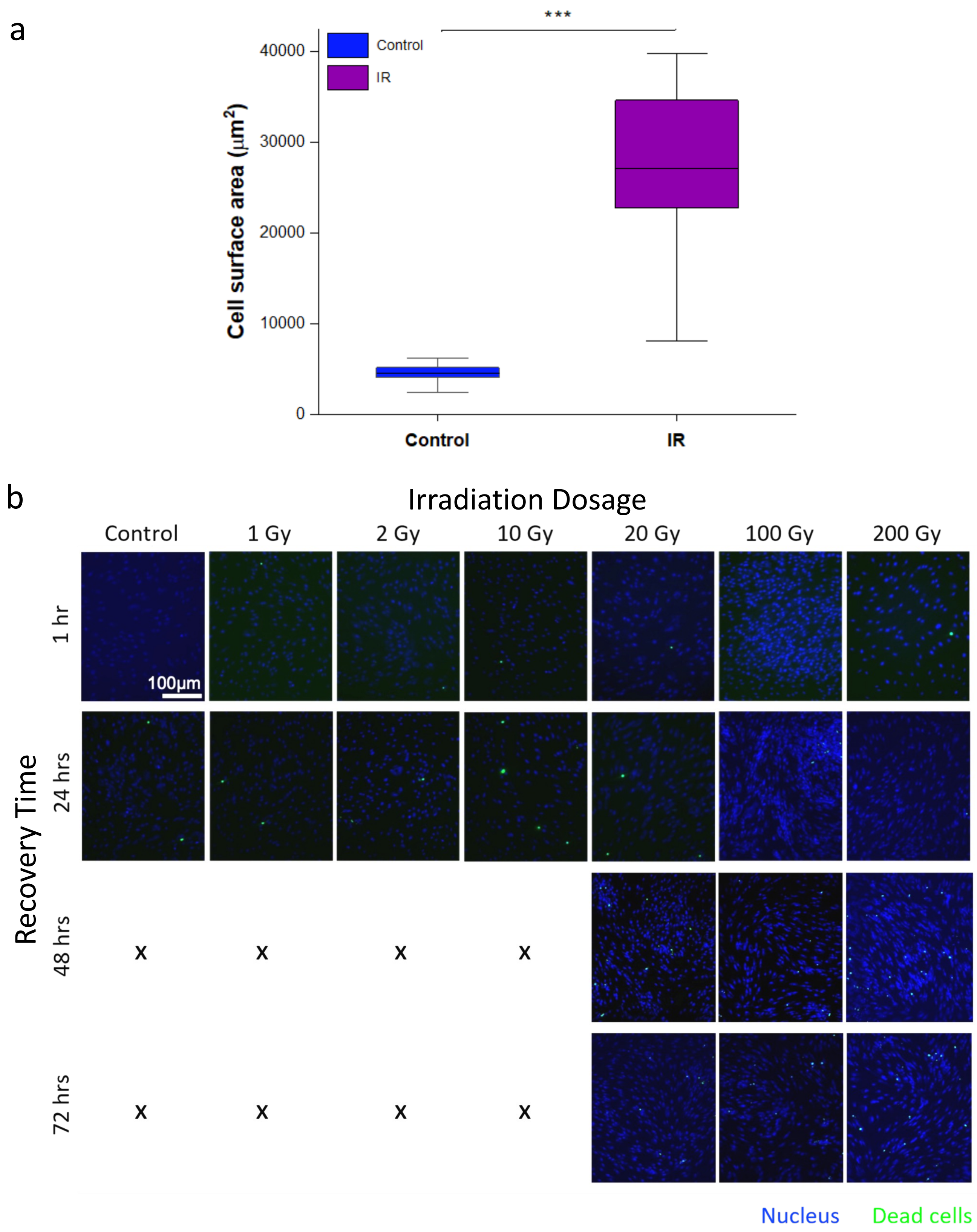

Figure S8. Effects of irradiation on cell morphology and viability. (a) Box and whisker plot illustrating the area of single cells for control and IR treated cells. Phase-contrast images were pooled to outline ten single cells for each condition. Representative images are shown in Figure 3b. Cell area was measured in pixel with ImageJ. Irradiation-treated (IR) MSCs (purple) have significant higher pixel surface area compared to control group (blue). **(b)** Live-dead staining of X-ray treated adMSCs with different recovery periods. In all tested conditions, adMSCs were exposed to various dosages of X-ray irradiation for under one hour. Different recovery times were tested to allow the cells time to morphologically respond to the treatment. Cells were ~95% viable up to 8 days following treatment. Green cells represent dead cells while blue cells represent live nuclei. Scale bar 100 μm.

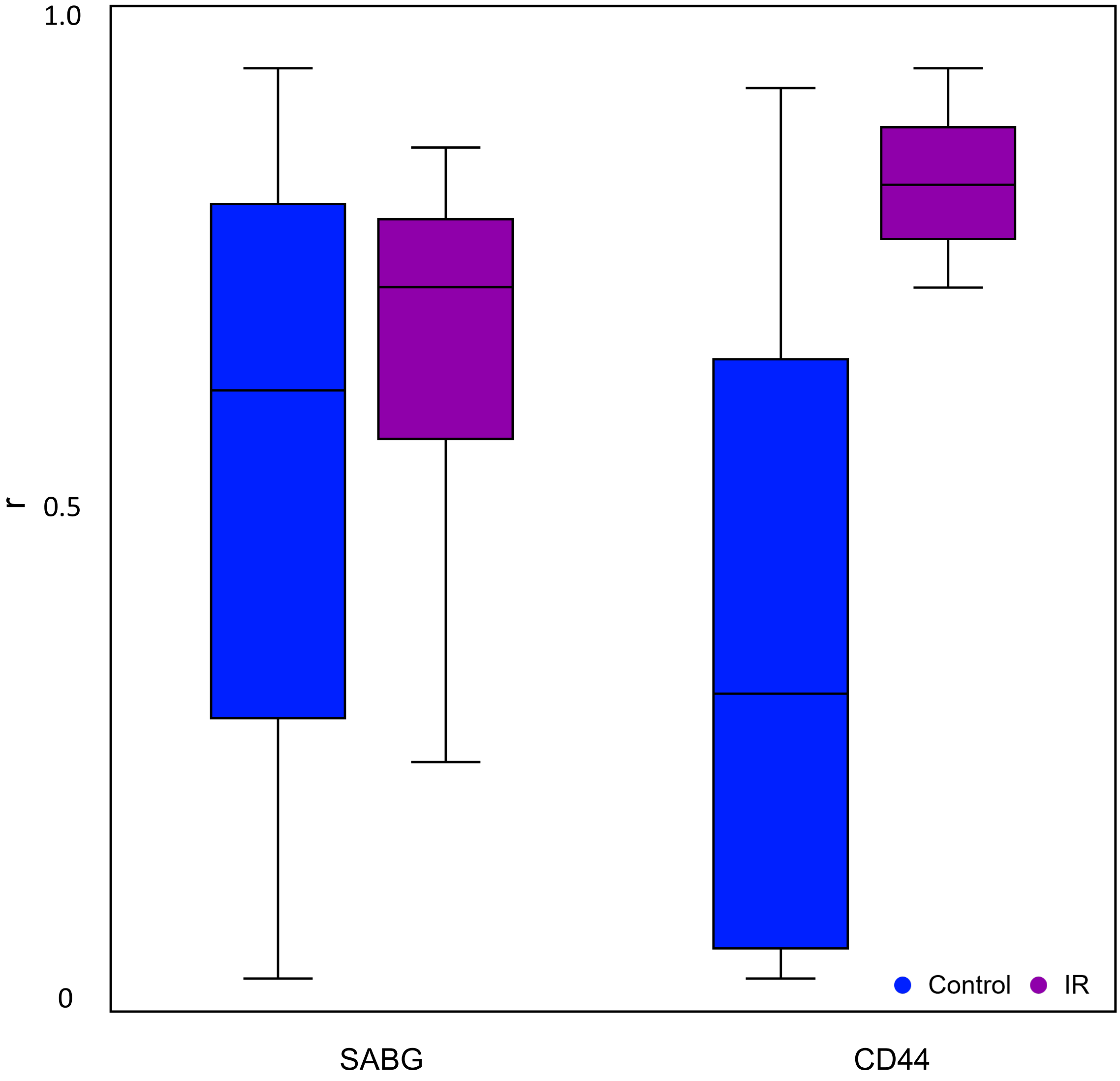

Figue S9. Pearson correlation coefficient between prediction and target for irradiated cells. Several phase contrast and immunofluorescence images of SABG and CD44 were pooled for analysis for both control MSCs and irradiation-treated (IR) MSCs. Representative phase contrast images are shown in Fig. 3b. Phase contrast images were inputted into a trained AI model for marker expression prediction. MSCs in AI-predicted images and the same MSCs in corresponding immunofluorescence images were individually outlined. Pearson correlation coefficient comparing these single cell MSC comparisons of CD44 and SABG in 30 control MSCs and 30 IR MSCs are shown.

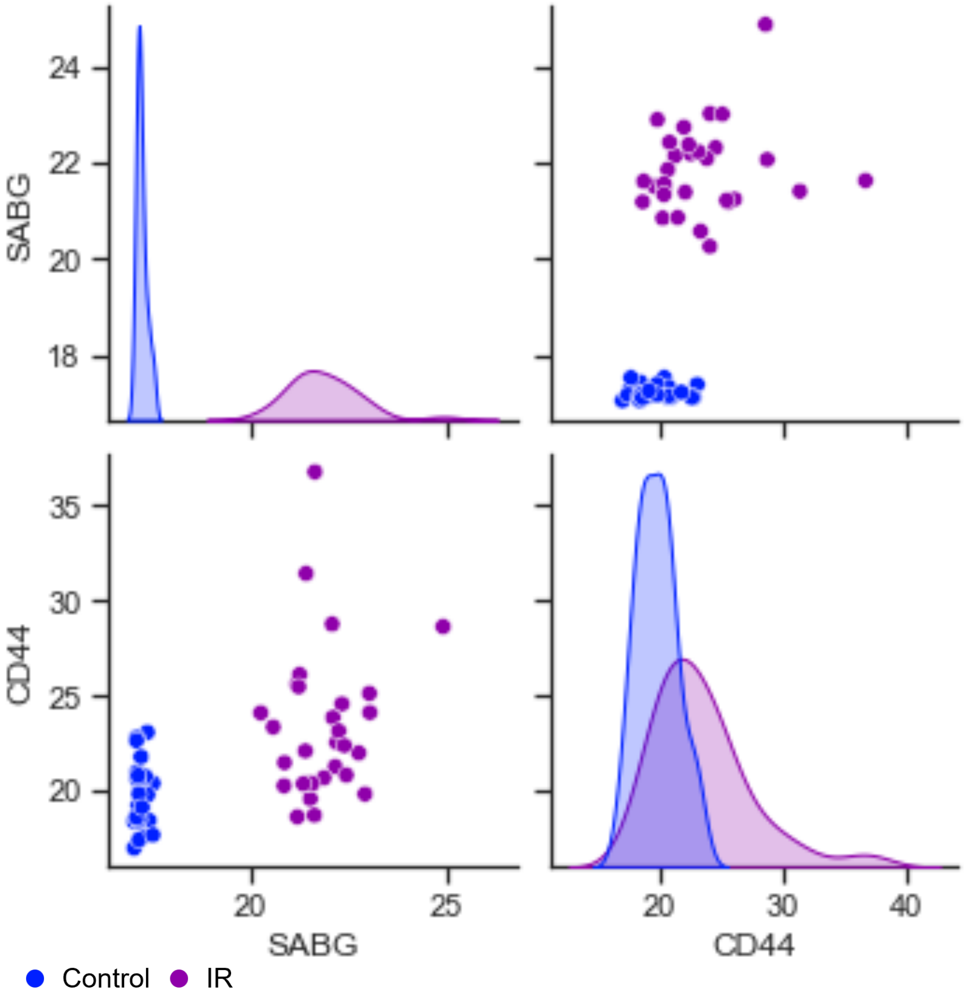

Figure S10. Irradiation bivariate plots. Several phase contrast images for both control MSCs and irradiation-treated (IR) MSCs were pooled for analysis. Representative phase contrast images are shown in Fig. 3b. Images were inputted into AI model for marker expression prediction. MSCs in AI-predicted images were individually outlined. Bivariate plot represents the AI model predicted expression of SABG and CD44 in 30 control MSCs and 30 IR MSCs. In these scatter plots, one data point represents one cell. Histograms demonstrate intensity distributions for each respective condition. These results confirm the distinct separation between IR-treated and untreated samples.

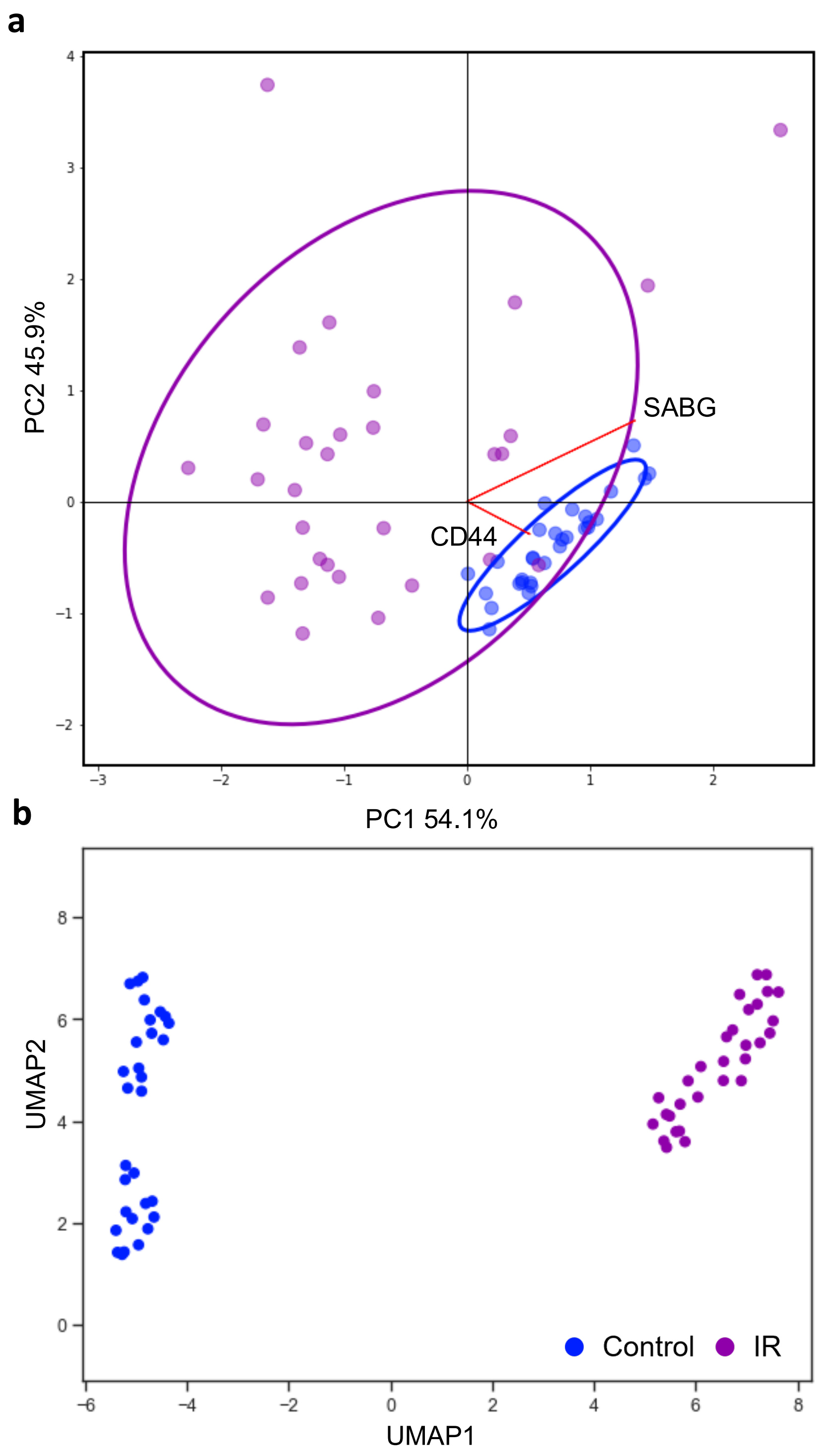

Figure S11. Irradiation PCA biplot and UMAP. Several phase contrast images for both control MSCs and irradiation-treated (IR) MSCs were pooled for analysis. Representative phase contrast images are shown in Fig. 3b. Images were inputted into AI model for marker expression prediction. MSCs in AI-predicted images were individually outlined. PCA biplot (**a**) and UMAP (**b**) represent the AI model predicted expression of CD44 and SABG in 30 control MSCs and 30 IR MSCs. 95% confidence ellipse is shown in PCA biplot. PCA biplot and UMAP demonstrate a clear distinction between control MSCs and IR MSCs.

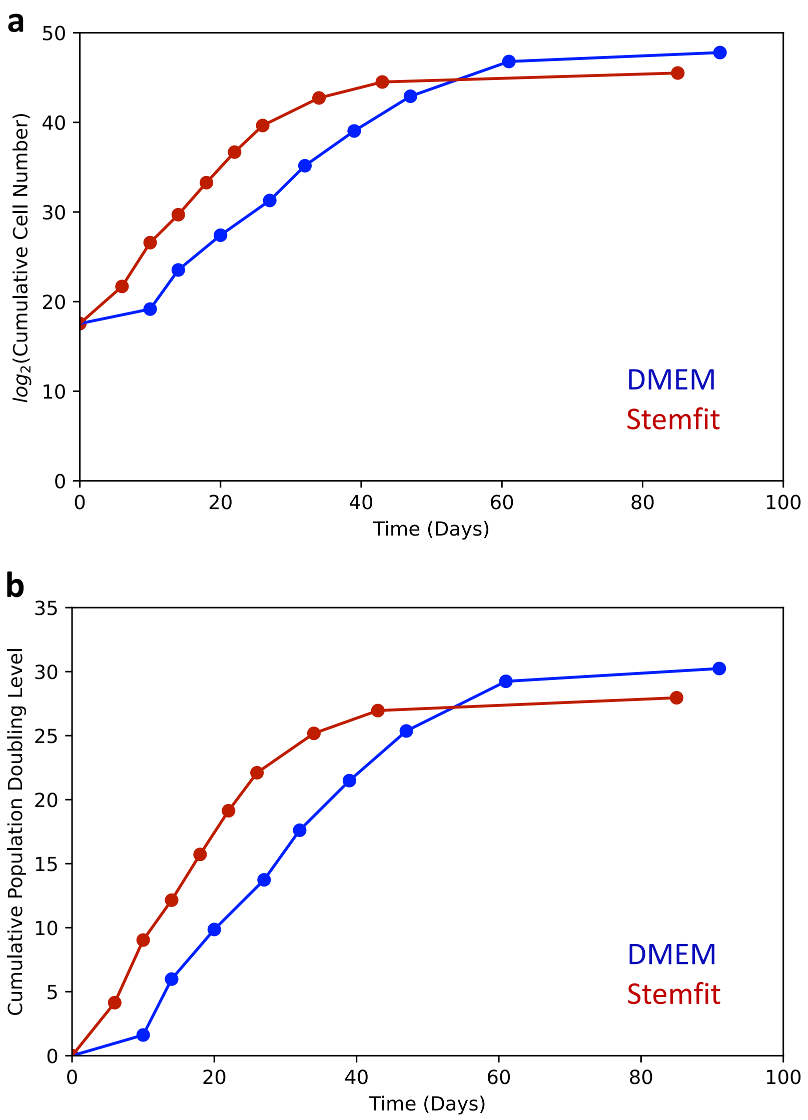

Figure S12. Inducing replicative senescence via cell passaging. MSCs were cultured from passage 2 to passage 10 in order to investigate the efficacy of our AI model in assessing the extracellular marker expression for replicative senescence in MSCs. We recorded the cumulative cell number (a) and cumulative population doubling (b), in which each point on the timeline represents cell passaging. As shown, both MSCs cultured in DMEM and MSCs cultured in Stemfit demonstrated a decline in growth rate as time increased. Both cultures of MSCs eventually reached a plateau in growth rate which signified senescence in the MSC cultures.

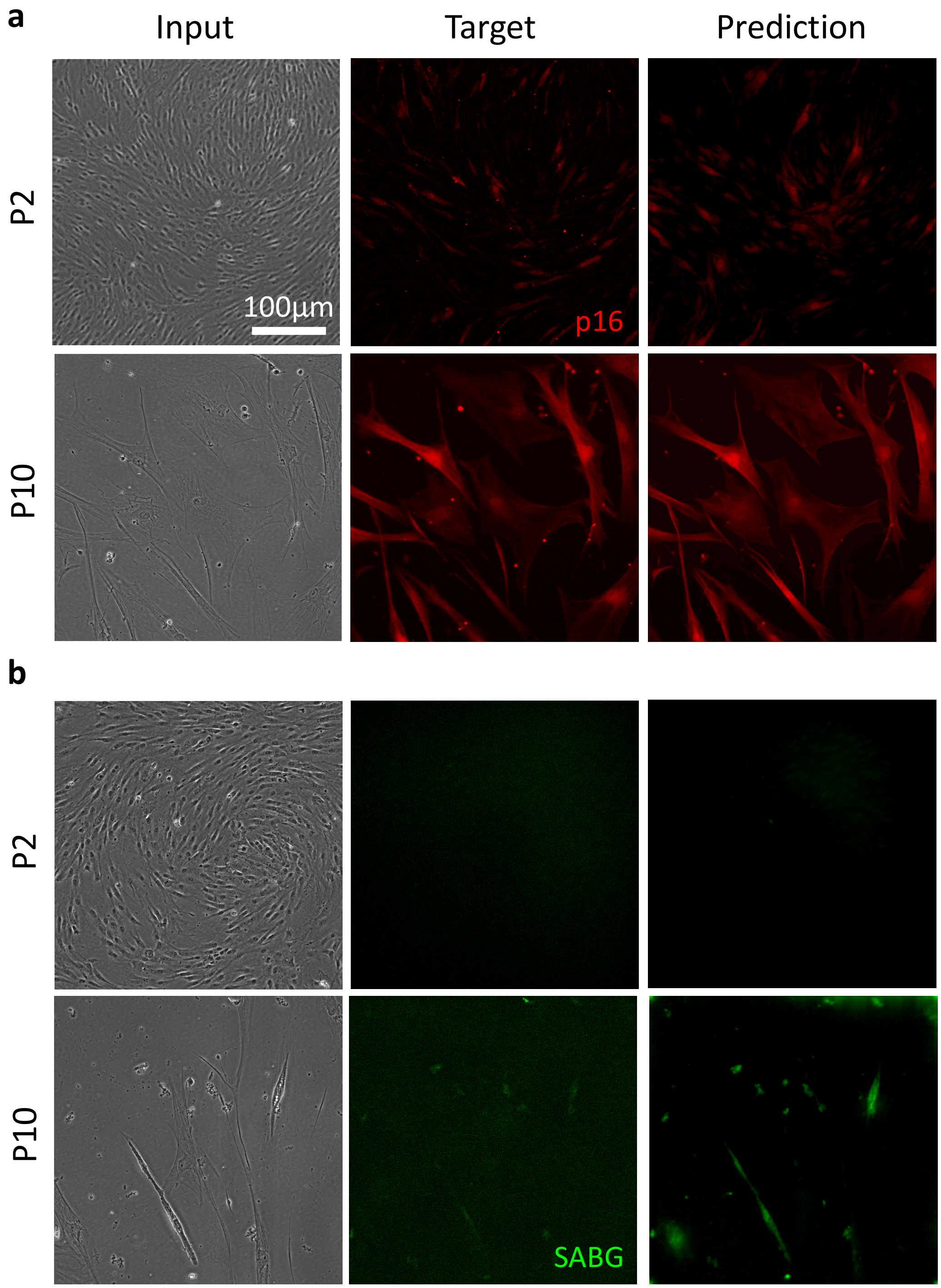

Figure S13. Replicative senescence in bmMSCs cultured in Stemfit. Left to Right: Phase-contrast (Input), antibody-stained immunofluorescence (Target), and ML-produced immunofluorescence images (Prediction). Top to Bottom: Passage 2 MSCs and passage 10 MSCs. (**a**) p16. (**b**) SABG. Senescence markers p16 and SABG get upregulated as more subculturing (cell passages) is performed. Scale bar for (**a**) and (**b**) is 100 µm.

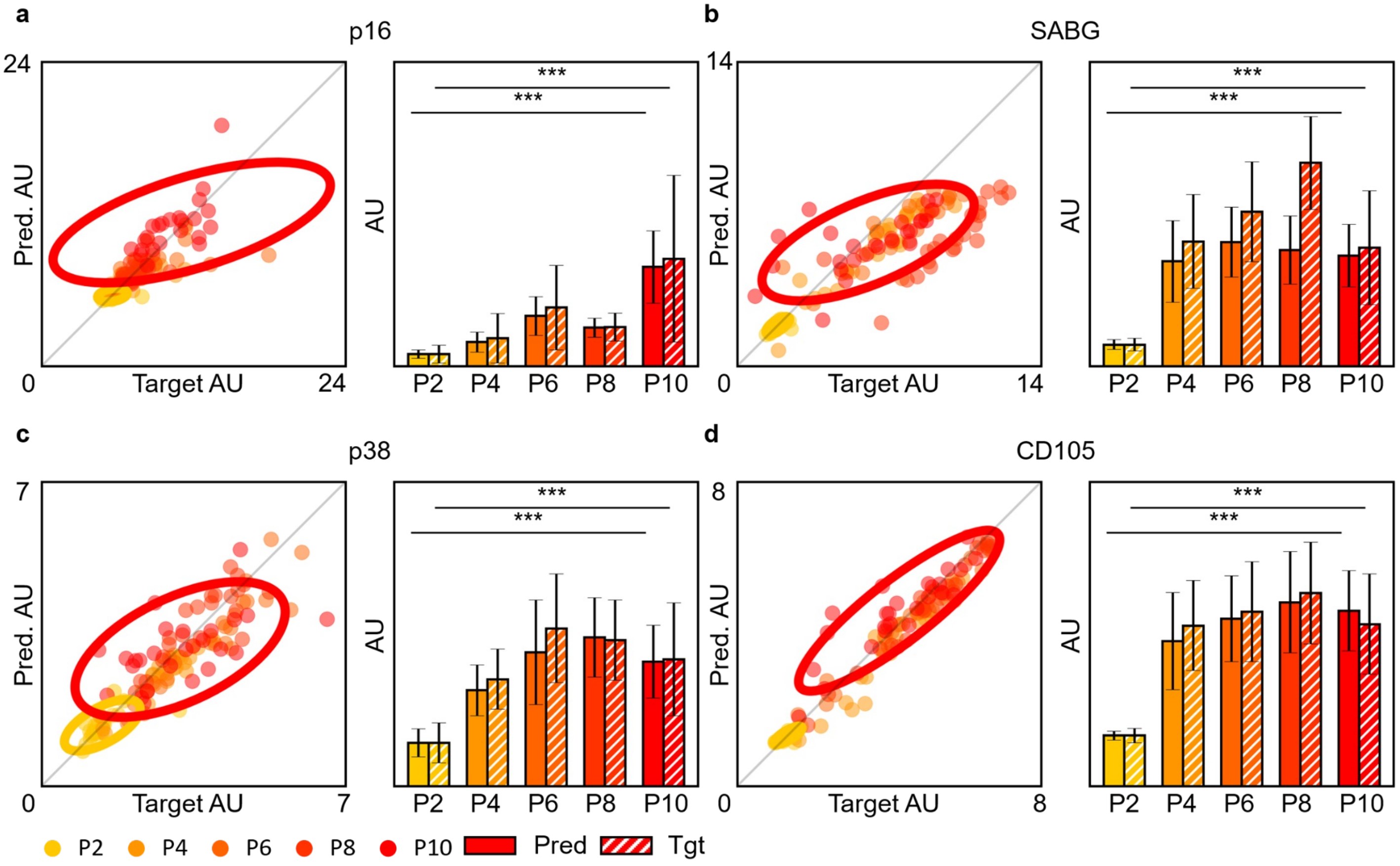

Figure S14. Replicative senescence marker quantification for bmMSCs cultured using Stemfit. (a) p16. (b) SABG. (c) p38. (d) CD105. Scatter plots demonstrate a strong positive correlation between target and prediction images for passage 2 MSCs cultured in Stemfit to passage 10 MSCs cultured in Stemfit stained for p16, SABG, p38, and CD105. Bar charts demonstrate a corresponding significant difference in p16, SABG, p38, and CD105 between passage 2 MSCs cultured in Stemfit and passage 10 MSCs cultured in Stemfit for prediction and target. N.S. not significant; * $p < 0.05$; ** $p < 0.001$; *** $p < 0.0001$.

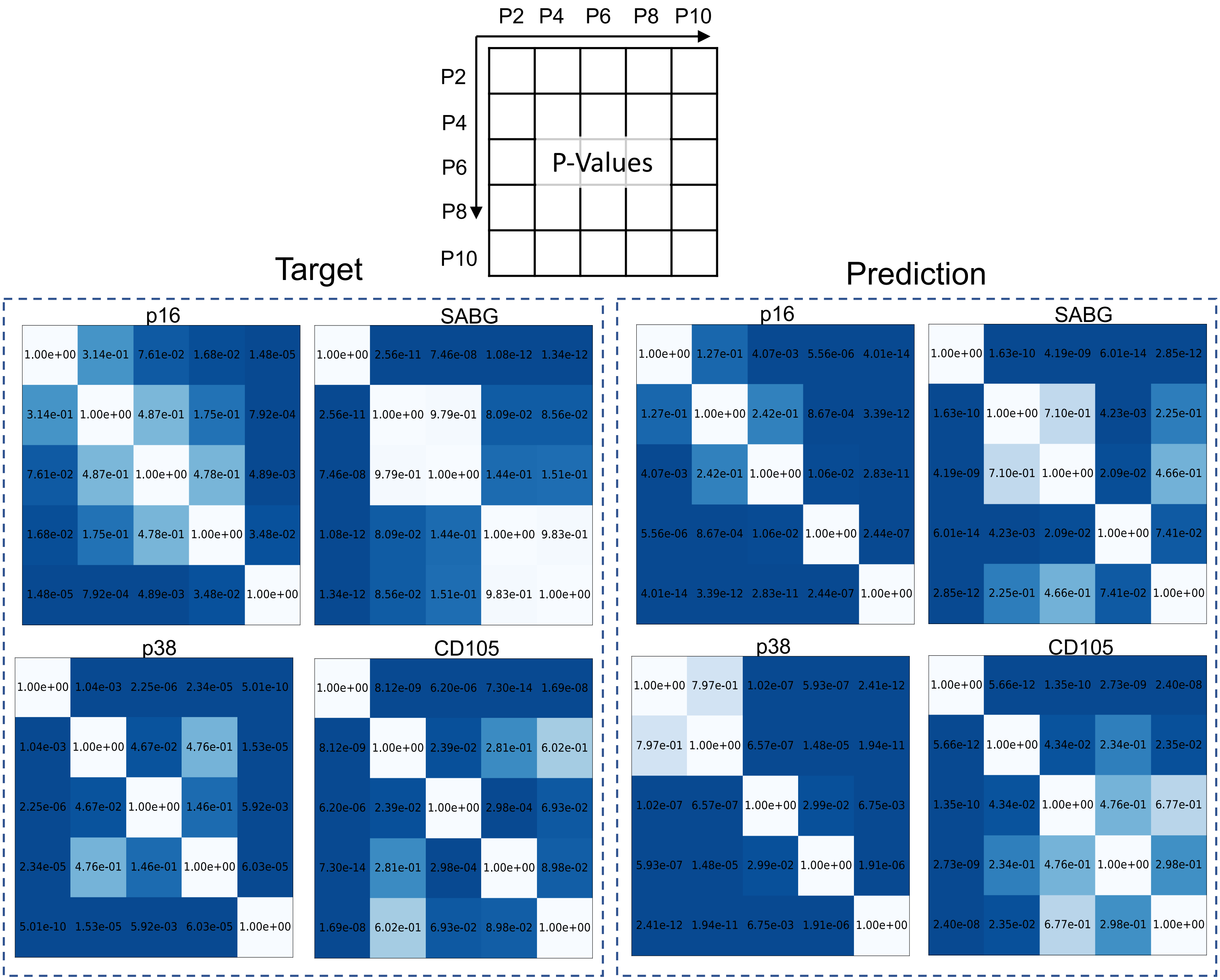

Figure S15. T-test of senescence marker expression levels in replicative MSC senescence. Matrices showing the marker expression level p-values for all pairwise passage combinations for both target (left) and predictions (right).

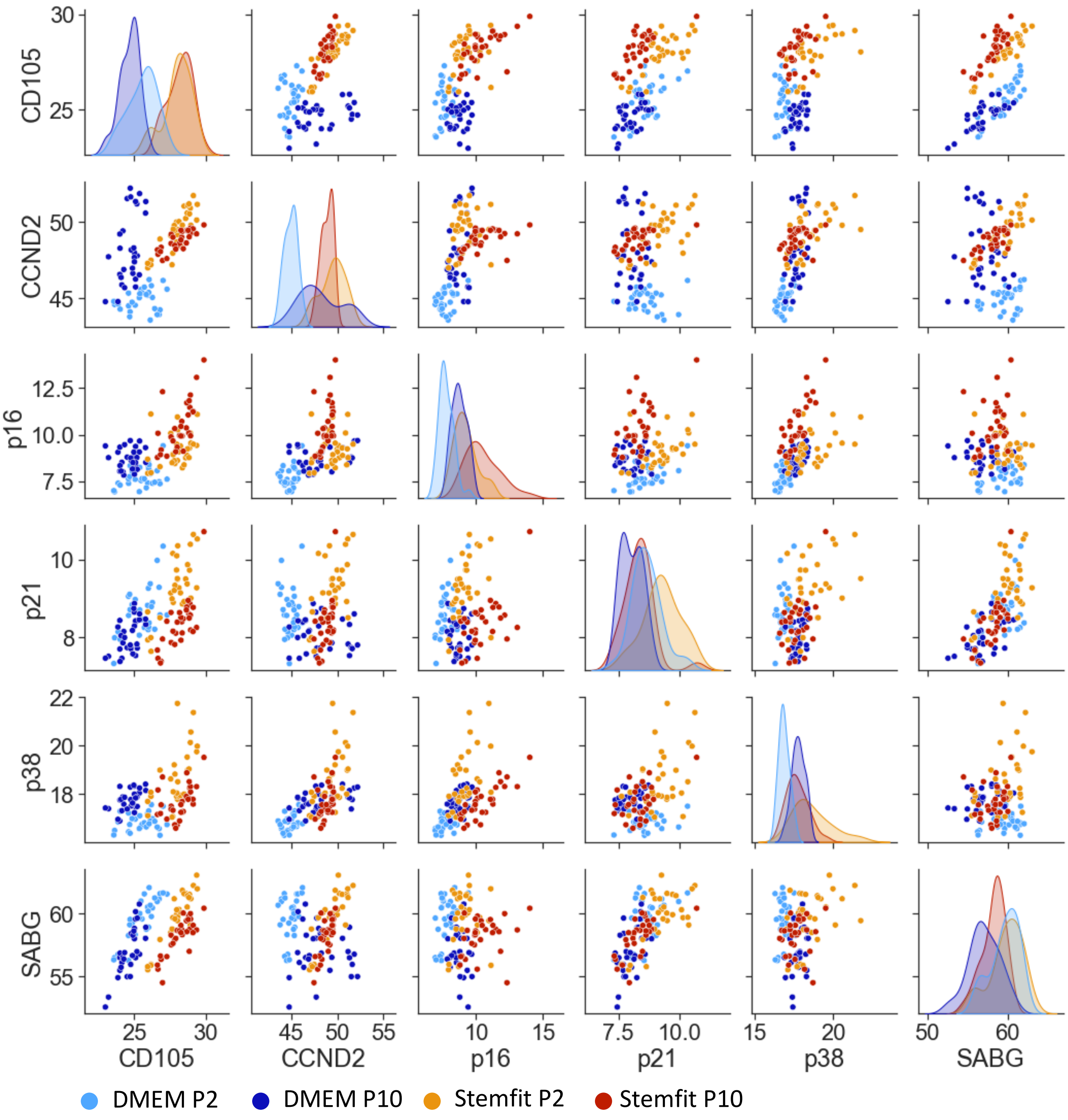

Figure S16. Replicative Senescence Bivariate Plots. Phase contrast images were taken for passage 2 MSCs cultured in DMEM media (DMEM P2), passage 10 MSCs cultured in DMEM media (DMEM P2), passage 2 MSCs cultured in Stemfit media (Stemfit P2), and passage 10 MSCs cultured in Stemfit media (Stemfit P10). Images were inputted into AI model for marker expression prediction. MSCs in AI-predicted images were individually outlined. Bivariate plot represents the AI model predicted expression of CD105, CCND2, p16, p21, p38, and SABG in 30 DMEM P2 MSCs, 30 DMEM P10 MSCs, 30 Stemfit P2 MSCs, and 30 Stemfit P10 MSCs.

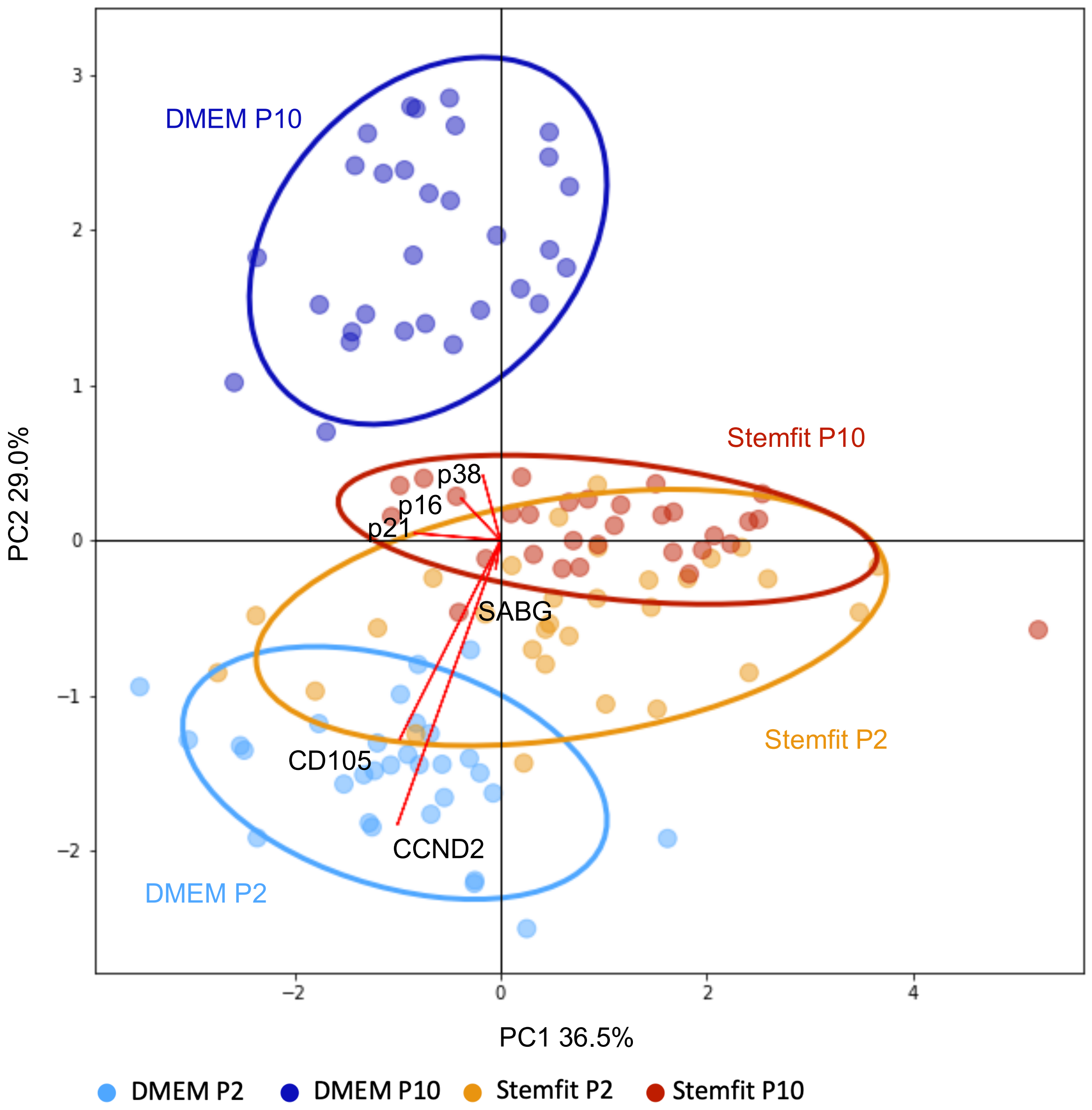

Figure S17. Replicative Senescence PCA biplot. Phase contrast images were taken for passage 2 MSCs cultured in DMEM media (DMEM P2), passage 10 MSCs cultured in DMEM media (DMEM P2), passage 2 MSCs cultured in Stemfit media (Stemfit P2), and passage 10 MSCs cultured in Stemfit media (Stemfit P10). Images were inputted into AI model for marker expression prediction. MSCs in AI-predicted images were individually outlined. PCA biplot represents the AI model predicted expression of CD105, CCND2, p16, p21, p38, and SABG in 30 DMEM P2 MSCs, 30 DMEM P10 MSCs, 30 Stemfit P2 MSCs, and 30 Stemfit P10 MSCs. 95% confidence ellipse is shown. PCA biplot demonstrates a clear distinction between passage for MSCs cultured in DMEM, however PCA biplot does not demonstrate a clear distinction between passage for MSCs cultured in Stemfit.
